# *opynfield*: An Open-Source Python Package for the Analysis of Open Field Exploration Data

**DOI:** 10.1007/s12021-025-09753-2

**Published:** 2025-12-10

**Authors:** Ellen McMullen, Miguel de la Flor, Gemunu Gunaratne, Jason O’Connor, Gregg Roman

**Affiliations:** 1 Department of BioMolecular Sciences, School of Pharmacy, University of Mississippi, Oxford, MS USA; 2 Department of Pharmacology, University of Texas Health Science Center at San Antonio, San Antonio, TX USA; 3 Audie L. Murphy VA Hospital, South Texas Veterans Health System, San Antonio, TX USA; 4 Department of Physics, University of Houston, Houston, TX USA; 5 Geriatric Research, Education and Clinical Center, South Texas Veterans Health System, San Antonio, TX USA; 6 Present Address: Program in Ecology, Evolution, and Conservation Biology, University of Nevada, Reno, NV USA

**Keywords:** Exploration, Novelty, Habituation, Directional Persistence, Drosophila, Mice

## Abstract

The open field test is widely used in behavioral neuroscience, providing insights into exploration, anxiety, and the learning processes associated with habituation to novelty. Analyses of exploratory behaviors in open field areas rely heavily on movement changes over time. These activity measures are susceptible to confounds from group differences in locomotor abilities and only provide an indirect measure of learning during exploration. Considerable effort has been placed on identifying additional measures of behavior that can better describe changes in exploration and habituation of novelty. Two measures for enhanced analysis of exploration are coverage and directional persistence (P_++_). Coverage measures the number of visits to segments of the arena boundary and represents the number of opportunities to habituate to the novelty of this boundary. P_++_ measures the probability of continued movement in the same direction, reflecting goal-directed exploration, which decreases as the animal habituates the novel arena. Our new Python package, *opynfield*, calculates coverage, P_++_, and activity measures from open field tracking data. We further introduce versions of coverage and the analysis of additional motion probabilities. The package includes new, in-depth statistical approaches and data visualizations. We demonstrate the applicability of *opynfield* using experiments with *Drosophila melanogaster* in which we (1) validate *opynfield*’s statistical tests, (2) substantiate coverage as a measure of novelty habituation, and (3) characterize behavioral differences in exploration. We also illustrate the utility of *opynfield* for analyzing rodent exploration by applying it to data from an experiment with *Mus musculus*. By leveraging full-density tracking data, *opynfield* facilitates a more nuanced understanding of exploration, potentially leading to improved insights into animal behavior and changes in learning, locomotor activity, and anxiety.

**Supplementary Information:**

The online version contains supplementary material available at https://doi.org/10.1007/s12021-025-09753-2.

## Introduction

Exploration plays a fundamental role in animal behavior and is crucial for their adaptive strategies. Exploratory behaviors can be classified by the nature of the stimuli eliciting them, the underlying motivation to explore, and how the animal explores its environs (Berlyne, 1966). *Specific exploration* is initiated by a lack of information about the immediate environment (Berlyne, 1960, 1966). Animals may seek information about novel stimuli from a distance, using perceptual exploration, or be motivated to examine the novel stimuli proximally. *Locomotor exploration* occurs when the animal moves around to explore its environment to gather information and become familiar with previously novel terrain (Berlyne, 1960). Ultimately, the animal learns and familiarizes itself with the objects and habituates to the novelty. This loss of novelty ultimately leads to the cessation of specific locomotor exploration (Soibam et al., 2013, 2014).

The novel open field test has been widely adopted for laboratory studies of exploration, anxiety-like behaviors, and the effects of xenobiotic compounds, using a variety of model organisms (Colomb & Brembs, 2014; Gould et al., 2009; Prut & Belzung, 2003; Walsh & Cummins, 1976). In these experiments, animals are placed in an empty, unfamiliar arena, and their movements are recorded as they explore (Walsh & Cummins, 1976). Studies of open field exploration have been facilitated by the development of software tools for tracking the position of animals over time in an open field assay. Commonly employed trackers include Ethovision (Wageningen, the Netherlands), Any-maze (Wood Dale, IL, USA), and BuriTrack (Colomb & Brembs, 2014), which can be utilized in real-time or to extract coordinates from pre-recorded videos. Typically, researchers analyze raw tracking coordinates by calculating activity levels, which are quantified as the distance moved by the animal at each time step. Activity levels are often binned into minute-long periods to assess changes in activity, serving as a proxy for exploration. Additional measures of the animal’s movement within the arena have also been employed to extract characteristics of their behavior (Choleris et al., 2001; Sturman et al., 2018).

The open field test has been used in *Drosophila melanogaster* to identify genes required for exploration and to better understand the learning processes involved in the habituation of novelty (de la Flor et al., 2017; Liu et al., 2007; Soibam et al., 2012a, 2013, 2014). *Drosophila* primarily use locomotor exploration to visually learn the arena boundary through proximal visits to each segment of the boundary (Liu et al., 2007; Soibam et al., 2012a). To better understand how the fly learns during exploration, the measurement coverage*,* a function of the number of visits to segments of the arena boundary, was developed (Soibam et al., 2014). Each visit to a boundary segment represents a learning opportunity (i.e., a training trial), and the rate of learning depends on the number of visits to each segment required to habituate the arena’s novelty. Hence, differences in learning during exploration may be better captured as a decrease in activity as a function of coverage rather than as a function of time, especially when the genotypes or experimental treatments differ in their movement or exploration strategies. While exploring, *Drosophila* also have a high probability for directional persistence as opposed to stopping or reversing course, which we quantify by a probability, P_++_ (Soibam et al., 2013), which decays with exploration and is likely a measure of an intentional movement, driven by the motivation to become familiar with the arena boundary (Soibam et al., 2012b, 2013, 2014). Hence, coverage and P_++_ can provide more comprehensive information on novelty habituation for animals with differences in locomotor activity (Soibam et al., 2014). Nevertheless, coverage and P_++_ do not have dedicated analysis tools, which has likely inhibited their widespread adoption for open field analysis.

In response to these challenges, we introduce *opynfield*, a novel software package developed to analyze coverage and directional persistence using open field data. The *opynfield* package leverages full density tracking data to calculate complex measures of exploration. It also facilitates in-depth statistical analyses and data visualization, providing a comprehensive platform for investigating exploration behavior in *Drosophila* species or other model organisms.

## Software Design and Implementation

### Overview

The *opynfield* package is written in the open-source programming language Python. The package is organized into seven main modules, covering configuration, data input, behavioral measure calculation, behavioral measure summary, model fitting, statistical tests, and plotting. Each module addresses a specific aspect of the analysis pipeline, allowing users to explore and interpret complex behavioral data. The following sections detail technical aspects of each module, but users are directed to the tutorial page for a more user-friendly introduction to performing an analysis with *opynfield*.

### Module 1: Configuration

The configuration module of *opynfield* (*config*) manages user inputs and essential analysis parameters using five primary data classes. Users can configure tracking data specifications, arena details, and analysis preferences, among other details. This module allows users to customize settings based on experimental needs, increasing the flexibility of the package.

The *UserInput* data-class sets the crucial parameters of an open field experiment, such as experimental groups and arena sizes, as well as user preferences for displaying progress updates and saving results during an *opynfield* run. It includes nine mandatory parameters, as well as four optional ones that can be left to their default values. Important inputs include *groups_and_types,* which specifies the experimental groups to be analyzed and the tracking software with which they were recorded, *arena_radius_cm,* which specifies the size of the arena used, and *sample_freq,* which specifies the recordings’ frame rate. Extensive documentation for all parameters is available on the *opynfield* GitHub page (see Availability), but *edge_dist_cm*, *time_bin_size*, and *inactivity_threshold* require further explanation here.

Because *Drosophila* species spend most of their time in the arena close to the boundary, certain exploratory measures are only calculated for time spent in this edge region. The user input *edge_dist_cm* determines the distance from the boundary considered the edge region. For *Drosophila* species, this is typically one centimeter or less. If a user does not want to restrict analysis to the edge of the arena, setting *edge_dist_cm* to the arena radius will eliminate this restriction.

The parameters *time_bin_size* and *inactivity_threshold* together address the problem of body wobble or tracking imprecision. The *time_bin_size* parameter is used to reduce the effective sampling frequency and indicates, in seconds, the desired time between consecutive points for analysis (one over the desired frequency). This allows the user to set an empirically informed *inactivity_threshold*, which determines the size of a step required to be considered non-zero. These settings should be considered together with the biology of the test subject to reduce tracking artifacts while preserving as much data as possible.

The *CoverageAsymptote* data class permits users to customize the models that are used to fit a relationship between time and coverage. This model is necessary to extract the asymptote value used to scale coverage in some analyses. The *f_name* field designates the curve fitting function. Several predetermined functions in the *config* module are available, or a user-defined function can be specified.

The *fixed_exponential* or asymptotic increase function (Eq. 1) is appropriate for *Drosophila* data, where coverage starts at zero, and asymptotically increases to a value that we interpret as the number of learning trials required to completely habituate to the arena. The value of *asymptote_param* specifies which parameter in the provided functions indicates the asymptote, in this case, the zeroth, *a*, while *asymptote_sign* indicates whether it is positive or negative. Users can further adjust the curve-fitting process through *initial_parameters*, *parameter_bounds*, and *max_f_eval*. This allows researchers to tailor the curve fitting to their specific experimental contexts, increasing the reliability of asymptote identification.

1 $$y=a({e}^{bx}-1)$$

Next, the *ModelSpecification* data class determines the functions used to model the relationships between all other behavioral measures. Each *ModelSpecification* instance contains *axes* that indicate independent and dependent variables, and a *model* that indicates the functional form to be used. The *model* includes additional associated parameters to specify initial parameters and bounds, and *display_parts,* which breaks up the equation into pieces that can be combined with the fit parameters to specify the best-fit function. A *ModelSpecification* instance must be created for each x and y variable pair to be examined, so the utility function *set_up_fits* automates the creation of these objects with the default *ModelSpecification* for each pair (based on *Drosophila* data) and organizes them into a dictionary. Users can then edit the *ModelSpecification* object for any of the variable pairs that they wish. Further details about choosing model forms can be found in the model fitting section.

Aesthetic customizations for the final plots are controlled by the *PlotSettings* data class. While *group_colors* is mandatory, allowing users to assign unique colors to each experimental group, other settings are optional. The optional settings can be customized to change marker sizes, include (or exclude) specific layers of the plots (such as model equations and error bars), and dictate which plots should be displayed or saved to files. As these settings primarily affect aesthetics, they will not be discussed further, but more details can be found in the package tutorial and documentation.

Finally, the *Defaults* data class contains other values that a typical user would not want to adjust for each experiment. For example, *node_size* determines how the arena is segmented for coverage calculation. The *node_size* parameter and other impactful settings are discussed alongside the measures that they influence. Additionally, *Defaults* empowers users to toggle off specific analyses or outputs to streamline use for different use cases.

### Module 2: Data Input

Due to the multitude of tracking software employed in the open field assay, such as Ethovision, BuriTrack, and Any-Maze, users may have tracking coordinate data in a variety of formats. The data input module (*readin*) is designed to accommodate the diverse file formats generated by these trackers, which allows for concurrent analysis of tracks from multiple file types. Additional documentation on supported file types and sample files of each format are available in the GitHub repository (see Availability).

The data input step loops through the file formats specified in the *UserInput* object. Raw data is then transformed into a file-type-specific track object for each specified file type. The subsequent standardization process results in the creation of a *Track* object, which contains essential information such as experimental group, x and y coordinates, recording timestamps, and tracker and arena details. This standardization ensures uniformity across tracks from all sources, aligning units, points of reference, and applying consistent smoothing procedures as needed. Because Ethovision tracks have a smoothing function automatically applied to the exported data, all other tracks should be smoothed to match. This smoothing is done automatically during data processing based on file type.

The data input step is orchestrated by the *run_all_tracks_and_types* function, which accepts the *UserInput* parameters as input. This function identifies the included file types and determines which experimental groups have tracks recorded in each file type. The function subsequently invokes *read_track_types* and transmits essential parameters to the file type-specific input functions. The specifics of these functions are detailed comprehensively in the documentation, but key procedural steps involve (1) invoking a graphical user interface for file selection, (2) parsing raw data, (3) extracting non-coordinate information such as group name or arena number, (4) retrieving x, y, and t coordinates, (5) unit conversion to centimeters and seconds, (6) zero-centering x and y coordinates, (7) smoothing data for consistency, (8) subsampling to the desired frequency, and (9) interpolating to address missing data points. The culmination of these steps results in storing processed data in a *Track* object, which provides the foundation for subsequent analyses.

### Module 3: Behavioral Measure Calculation

The behavioral measure calculation module (*calculate_measures*) extracts meaningful insights from tracking coordinates by calculating measures of exploration, including activity, coverage, and motion probabilities. Alternative versions of coverage and motion probabilities exist as well, allowing the user to choose an appropriate version for their specific project. The use of multiple measures provides a more complete understanding of the subject’s exploration by identifying specific behavioral changes responsible for changes in activity. The function *tracks_to_measures* coordinates the calculation of all measures for each *Track* object and generates a *StandardTrack* object, which consolidates information such as experimental group, coordinates, tracker, and arena details, and each behavioral measure.

Activity (ΔD) is the most fundamental metric of exploration. It is defined as the distance the subject travels between consecutive time points (Eq. 2). Note that *n* coordinates will have *n-1* “steps” between them. Activity at time *i* is defined as the step that begins at time *i* and ends at time *i* + *1.*

2 $$\Delta {D}_{i}=\sqrt{{({x}_{i+1}-{x}_{i})}^{2}+{({y}_{i+1}-{y}_{i})}^{2}}$$

Animals typically have higher activity at the beginning of the recording, when they are first introduced to the novel arena, and this activity decays over time. Animals in larger arenas also tend to have higher activity for more extended periods, as there is more space to explore. Therefore, exploration, as measured by activity, is a function of both space and the perception of novelty.

**Fig. 1:**
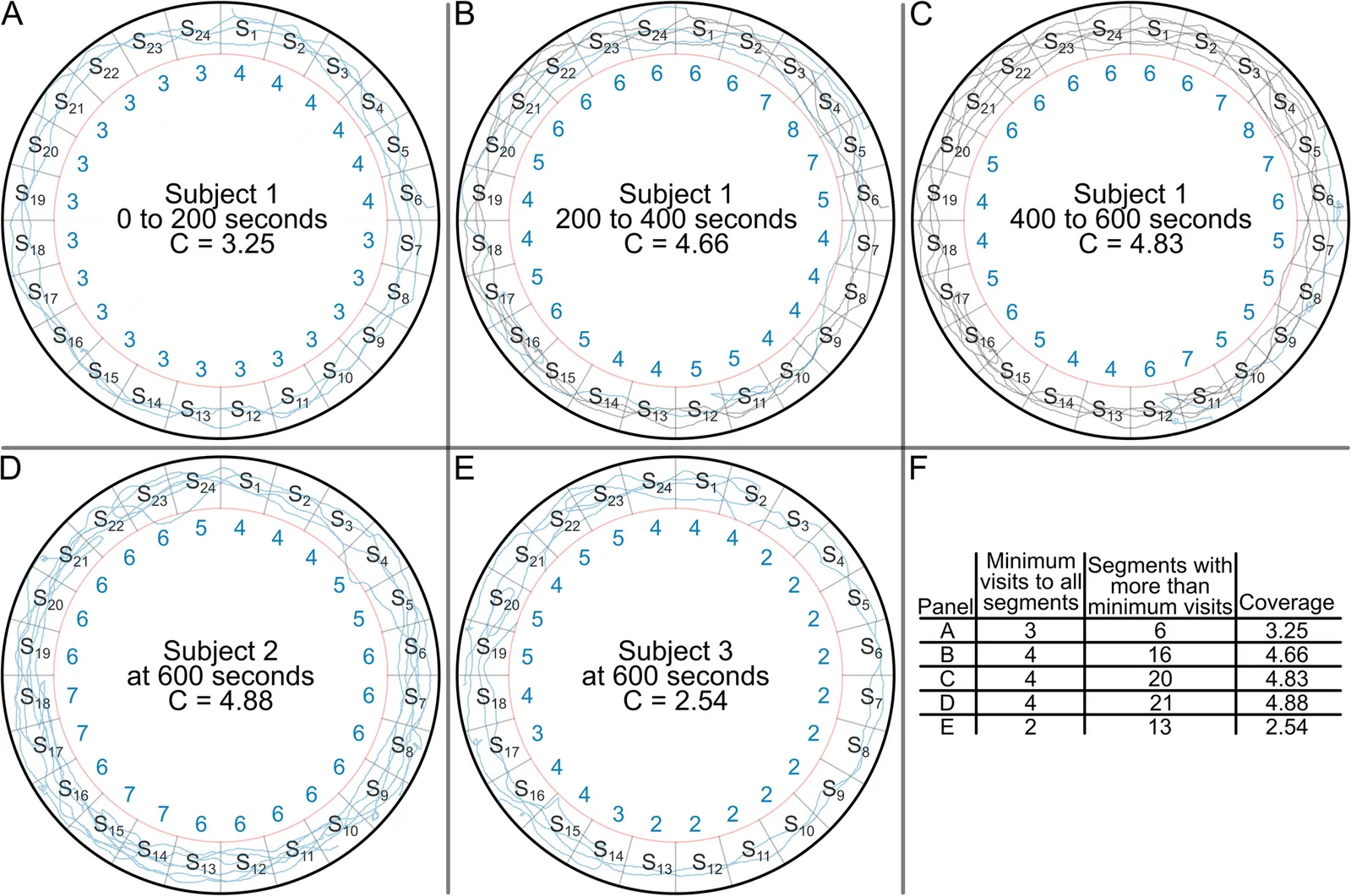
Prototypical examples of coverage. Throughout the course of an open field experiment, the subject explores the arena boundary. After dividing the arena into sectors (S_1_ to S_24_), we can count the number of visits made to each sector of the arena boundary and use the number of visits to calculate coverage. Each subject accumulates coverage over time. (**a**) A hypothetical subject, Subject 1, is tracked (blue trace) over the first 200 s of an experiment. The subject is first detected in sector 6 and makes three complete laps around the arena. Additionally, it travels from S_6_ to S_1_, making 4 total visits to those sectors. Thus, at t = 200 s, Subject 1 has v_min_ = 3, with 6 out of 24 sectors receiving more than v_min_ visits at that time. (**b**) The experiment continues to t = 400 s, and the subject makes more visits to each sector. The subject makes an additional complete lap, but doubles back several times, so that some sectors accumulate more visits than others. The number of visits made to each sector includes the visits accounted for previously (t = 0 to 200 s, gray trace), and the new visits made (t = 200 to 400 s, blue trace), which are considered together to count the number of visits to each sector. Then, coverage at t = 400 s is calculated from v_min_ = 4, with 16/24 sectors receiving more than v_min_ visits. (**c**) The experiment continues until t = 600 s, and the subject travels from S_5_ to S_12_, doubling back several times. Previous visits (t = 0 to 400 s, gray trace) and new visits (t = 400 to 600 s, blue trace) are considered together to count the total number of visits made to each sector. Coverage at t = 600 s is calculated from v_min_ = 4, with 20/24 sectors receiving more than v_min_ visits. When we calculate coverage at a finer timescale (typically every second, rather than every 200 s) and with more sectors (typically 3600, rather than 24), we can plot a nearly continuous function of how coverage accumulates over time. In addition to looking at how coverage accumulates over time within a given subject, we can also compare the total amount of coverage different subjects achieve throughout the course of an experiment. For example, Subject 2 (**d**) visits every sector at least 4 times, and visits 21 sectors more than 4 times, achieving a final coverage of 4.88. On the other hand, Subject 3 (**e**) only visits every sector at least 2 times, and visits 13 sectors more than 2 times, achieving a final coverage of 2.54. Thus, subject 2 requires approximately 2 × more learning opportunities to habituate to the novelty of the arena boundary than subject 3 does. If subjects 2 and 3 were part of the same experiment, this may represent genetic differences or effects of treatments. However, if they were run at different times, as a part of different experiments, it may represent differences in environmental conditions that influence the habituation processes, such as time of day or light levels. In panels **a**-**e**, blue numbers represent the number of visits made to the nearest sector. (**f**) These visits are used to calculate coverage by counting the minimum number of visits and the number of sectors with more than the minimum visits

Coverage, a metric developed to mitigate the arena-size-dependency inherent in activity, is introduced as a novelty-based measure of exploration. In *opynfield,* we employ a sector-based approach to calculate coverage, dividing the arena area into sectors and counting visits to each sector. Visits are not merely the number of recording points located inside each sector. An animal may linger in one sector for an extended period, but this still constitutes one visit. On the other hand, depending on the sampling frequency and sector size, an animal may not be recorded inside a sector that it necessarily would have passed through to travel between the sectors it was recorded in. The function *locate_bins* identifies the number of visits made to each bin over time, which the function *calculate_coverage* uses to compute the final measure as defined in Eq. 3. This equation shows how coverage (C) can be calculated at each time point (t) from the number of visits made to each sector by that time point. Here, v_min_(t) is the minimum number of visits made to any sector, such that all sectors at that time point have received a number of visits greater than or equal to v_min_(t). M represents the total number of sectors, so that the sum from i to M is iterating over all sectors. χ is the indicator function where χ[x] = {1 if x > 0, 0 otherwise}. v(i,t) is the number of visits made to sector i at time t. Thus, the argument v(i,t) – v_min_(t) is greater than 0 when sector i has received more visits than v_min_ at time t. In total, the summation counts the number of sectors that have received more than v_min_ visits, and this is divided by the total number of sectors, M. Thus, coverage is essentially the number of visits that every sector has received plus the fraction of sectors that have been visited more times than that. This calculation is further illustrated graphically (Fig. 1).

3 $$C\left(t\right)={v}_{\mathrm{min}}(t)+\frac{1}{M}\sum_{i=1}^{M}\chi [v\left(i,t\right)-{v}_{min}(t)])$$

For edge-dwelling animals such as *Drosophila* species, visits are only counted when they occur within a certain distance of the arena’s edge. This distance is controlled by the *UserInput* parameter, *edge_dist_cm*. The size of the sectors is defined by the *Defaults* parameter *node_size*, which determines the sector’s central angle in degrees, which must divide evenly into 360° to have equally sized sectors. While coverage is robust to changes in *node_size*, care should be taken to ensure the value results in sector arc lengths that make sense for the study subject (i.e., not orders of magnitude more or less than the subject’s body length). Because of these considerations, Cartesian coordinates (x and y) are converted to polar coordinates (r and theta), using the function *cartesian_to_polar*.

Coverage accurately measures the number of visits to each sector that an animal needs to learn or habituate to the novelty of that area. However, using raw coverage to examine activity or some other behavioral measure as a function of learning has limitations. The number of visits does not directly impact activity, but rather how much of the novelty habituation has been achieved by that time; when the animal has fully familiarized itself with its environment, it ceases specific exploration. Thus, *opynfield* calculates several re-scalings of coverage to use learning as a predictor of behavior. These alternative measures include percent coverage, percent of individual coverage asymptote (PICA), and percent of group coverage asymptote (PGCA).

Percent coverage simply takes an individual’s coverage vector and divides every value by the maximum coverage achieved during the recording. This normalization rescales the measure to be between 0 and 1, where the percent coverage value represents what fraction of novelty habituation has occurred. Percent coverage works well as a predictor of other behavioral measures when the animal reaches or nearly reaches the coverage required for full learning of the arena during recording.

If the recording time is too short for the animal to reach the coverage required to fully learn the arena (i.e., the time versus coverage relationship shows coverage is still increasing quickly and not reaching an asymptote), then the maximum coverage achieved during the recording is not a good measure of the animal’s coverage needed for full learning. Instead, PICA or PGCA may be more appropriate. In these cases, the coverage vector is rescaled by the predicted asymptote of the time versus coverage relationship. For PICA, the asymptote is calculated for each individual, while for PGCA, the group’s average coverage asymptote is used.

The functions *calculate_percent_coverage* and *calculate_pica* are used to calculate their respective measures as each *Track* is addressed. PGCA cannot be calculated until all *Track* objects have been converted to *StandardTrack* objects, at which point *tracks_to_measures* groups them by experimental group and calculates the group coverage asymptote with *calculate_group_coverage_asymptote*. At this point, the PGCA for each *StandardTrack* is calculated with *calculate_pgca*.

**Fig. 2:**
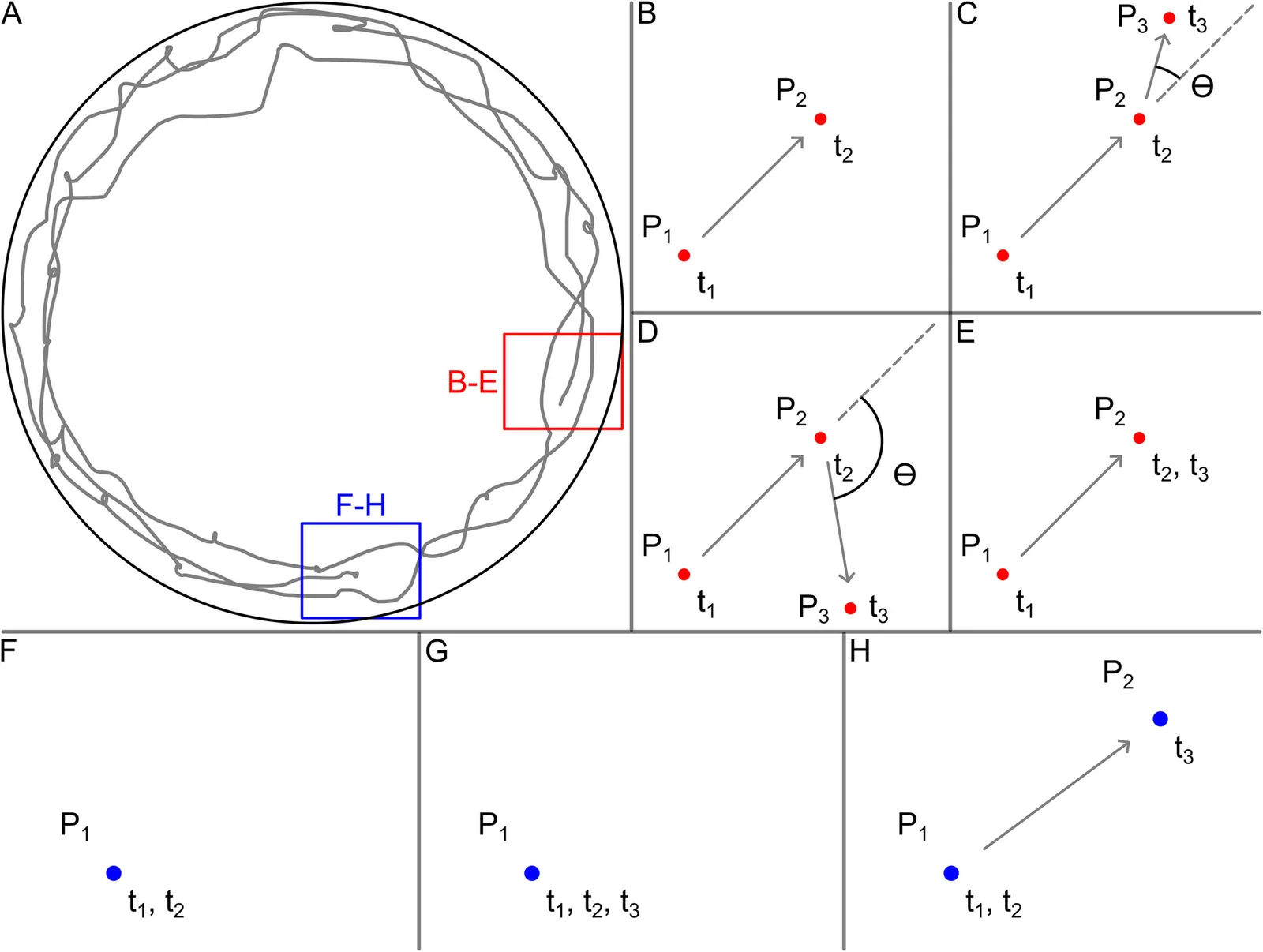
Prototypical examples of directional persistence behaviors. Throughout the course of an open field experiment, the subject explores the arena boundary. The overall exploratory path (**a**) can be broken down into step-by-step decisions. At the beginning of an experiment (red box, panel a), *Drosophila* exhibit high directional persistence. (**b**) Following a step forward from point P_1_ at time t_1_ to P_2_ at t_2_, the subject’s next step can be classified into three categories. (**c**) The subject can take another step in the same direction (❘ϴ❘ ≤ 90°) to P_3_ at t_3_, contributing to P_++_. (**d**) The subject can take a step in the reverse direction (❘ϴ❘ > 90°) to P_3_ at t_3_, contributing to P_+-_. (**e**) The subject can stop and remain at P_2_ until t_3_, contributing to P_+0_. (**f**) Later in an experiment (blue box, panel a), *Drosophila* exhibit less directional persistence and more rest. Following a rest, where the subject remains at P_1_ from t_1_ to t_2_, the subject’s next step can be classified into two categories. (**g**) The subject can stay at P_1_ until t_3_, contributing to P_00_. (**h**) The subject can take a step in any direction to P_2_ at t_3_, contributing to P_0+_

The module culminates with the computation of motion probabilities, which measure the directional persistence of test subjects. These probabilities, including P_++_, P_+-_, P_+0_, P_0+_, and P_00_ (Eqs. 4–8), evaluate the likelihood of specific turns or decisions between consecutive steps (Fig. 2). By accounting for directional changes, these probabilities contribute to a more nuanced understanding of exploratory drive and how it differs from random motion in the arena. These measures are calculated for a population rather than for individuals.

4 $${P}_{++}\left(n\right)=Prob(\Delta {D}_{i}>0|\Delta {D}_{i-1}>0)$$

5 $${P}_{+-}\left(n\right)=Prob(\Delta {D}_{i}<0|\Delta {D}_{i-1}>0)$$

6 $${P}_{+0}\left(n\right)=Prob(\Delta {D}_{i}=0|\Delta {D}_{i-1}>0)$$

7 $${P}_{0+}\left(n\right)=Prob(\Delta {D}_{i}>0|\Delta {D}_{i-1}=0)$$

8 $${P}_{00}\left(n\right)=Prob(\Delta {D}_{i}=0|\Delta {D}_{i-1}=0)$$

In these equations, the sign of ΔD is taken relative to the previous step. This means that the motion probabilities are calculated based on the angle between two consecutive steps (three consecutive tracking points). When the magnitude of an angle is less than 90°, that turn is considered positive or in the same direction as the previous step, while a turn with an angle greater than 90° is considered negative or in the opposite direction as the previous step. Undefined turns occur when one or both steps have a ΔD = 0, in which case P_+0_, P_0+,_ and P_00_ are determined by step length alone. Since the motion probabilities are contingent on their initial condition being met (a step occurring for P_++_, P_+-_, and P_+0_, or a rest occurring for P_0+_ and P_00_), some motion probabilities will be very rare to observe, especially P_00_. The function *turning_angle* uses the law of cosines to determine the angle between consecutive steps. These angles are taken together with activity to compute the motion probabilities. Note that for *n* coordinates, there are *n-1* steps and *n-2* turns or decisions. A decision or turn taken at time *i* occurs between step *i-1* (time *i-1* to *i*) and step *i* (time *i* to *i* + *1*). Additionally, calculation shortcuts can be made due to the relationships among the motion probabilities given in Eqs. 9 and 10.

9 $${P}_{++}+{P}_{+-}+{P}_{+0}=1$$

10 $${P}_{0+}+{P}_{00}=1$$

The function *motion_probabilities* first determines what type of turn or decision was made by each individual, generating an array of ones and zeros for each measure. Taking P_++_ as an example, a one in this array would mean that at that time point, the animal had just completed a step and was initiating a step in approximately the same direction, while a zero would mean it either had not completed a previous step (was at rest; P_0+_ or P_00_), or did complete a step, but was initiating a step in the opposite direction (P_+-_) or was initiating a rest (P_+0_). Next, the group’s motion probabilities are calculated with *motion_probabilities_given_previous*. For example, P_00_ at time *i* is the number of individuals with a 1 in their individual P_00_ array at time *i*, divided by the number of individuals with a 1 in either their P_00_ or P_0+_ array at time *i*. Thus, we set up an array with 1 where P_00_ occurred, 0 where P_00_ could have occurred but didn’t (i.e., P_0+_ occurred), and NaN elsewhere.

These measures may apply differently in other study organisms, so two additional versions were developed. The original version of the motion probabilities described above is designated motion probabilities given previous (e.g., P_+-Given+_) elsewhere in the documentation to differentiate it from motion probabilities given any and raw motion probabilities. Motion probabilities given any (e.g., P_0+GivenAny_) are calculated similarly. For example, this P_00_ at time point *i* is the number of individuals that had a 1 in their P_00_ array at time *i*, divided by the number of individuals that had a 1 in their P_++_, P_+-_, P_+0_, P_0+_, or P_00_ array at time *i*. In this case, Eqs. 4, 5, 6, 7, 8, 9 and 10 do not hold, but instead the relationship is found in Eq. 11. This differs from raw motion probabilities because all versions of motion probabilities are calculated only in the edge region. This means that the sum of individuals that had a 1 in their P_++_, P_+-_, P_+0_, P_0+_, or P_00_ array at time *i* is not necessarily the total number of individuals in the group. Raw P_++_ would be calculated with the number of individuals in the group in the denominator.

11 $${P}_{++}+{P}_{+-}+{P}_{+0}+{P}_{0+}+{P}_{00}=1$$

### Module 4: Behavioral Measure Summary

Once these exploration metrics are calculated, the behavioral measure summary module (*summarize_measures*) synthesizes the individuals’ data into group-level insights. Additionally, the module produces outputs that can be used for external analysis.

The initial step in this module involves the *individual_measures_to_dfs* function, which compiles individual behavioral measures for each group into CSV files. Next, the module employs the function *all_group_averages* to calculate each relationship's group-level average and standard error of the mean (SEM). Group calculations are straightforward for temporal relationships, such as computing the mean activity at each time point, since all tracks are measured with the same sampling frequency. However, complexities arise for predictors like cover. For example, plotting average coverage against average activity would fail to capture the relationship seen in the individuals. Instead, this approach would show the average activity and coverage at a certain time point rather than the average activity at a certain coverage level. To address this, *opynfield* reverts to raw data for measures involving coverage. Tuples of coverage-activity pairs from all individuals in a group are aggregated, and the average activity values at specific coverage points are computed. To handle the continuous nature of coverage, bins or ranges of coverage values are introduced to group these tuples to be averaged. These bins are determined either by the number of tuples they contain (n_points) or by a set range size (n_bins). The n_points method performs better and is employed for coverage, PICA, and PGCA, while the n_bins method performs better and is used for percent coverage. Once each temporal and coverage-based relationship is summarized, a CSV file is produced for the average and SEM of each group.

### Module 5: Model Fitting

The model fitting module (*fit_models*) is used to reveal the relationships between exploratory measures at individual and group scales. The module calculates the parameters that optimally characterize these relationships, employing the functions specified in the earlier *ModelSpecification* objects. For instance, the time versus activity relationship in *Drosophila* species takes the form of an exponential decay (Eq. 12), where *a*, *b*, and *c* are parameters that govern different facets of the exploration process. The exponential decay form aligns with the observed behavior of *Drosophila* species, where activity starts at elevated levels and decreases asymptotically.

12 $$y=a{e}^{bx}+c$$

Due to the potential presence of outliers, *opynfield* employs a robust strategy to prevent the undue influence of outliers on the analysis. Instead of outright exclusion, which could potentially remove most of the data due to the large number of relationships being analyzed, the module employs parameter bounds based on the distribution of parameters observed among individuals in the same group during the naïve fit. The function *fit_all* performs the naïve fit, after which *find_fit_bounds* establishes parameter ranges, which span two standard deviations below to two standard deviations above the mean of each parameter. This bound-setting approach is modifiable through the optional *UserInput* parameter *bound_level* and provides a balanced means to address outliers without discarding useful data.

After the computation of parameter bounds, *opynfield* employs the *re_fit_all* function, which integrates these constraints to calculate the new best-fit parameters. The resultant values are exported as a CSV file for further analysis via the *format_params* function. These formatted individual parameters also set the stage for *opynfield*’s in-house statistical analyses.

In addition to the best-fit parameters for each individual, the group-level model parameters may also be of interest. The function *group_fit_all* leverages the previously calculated parameter bounds from individual fits and applies them to the model fitting process using the group-averaged data. The ensuing group parameters are formatted for future analysis by *format_group_params* and exported into CSV files.

### Model Choice

Due to the diversity of study organisms and experimental contexts that open field exploration can address, *opynfield* accommodates flexibility in the functional forms employed for model fitting. Table 1 outlines the six functional forms used in the sample analyses. The default *ModelSpecification* settings use the exponential decay model (Eq. 12) for activity, P_++_, and P_+0_, and the asymptotic increase model (Eq. 1) for coverage, P_+-_, P_+0_, and P_00_. Users can select a different pre-defined function based on known patterns of exploration or patterns observed in their data. For example, in the fourth sample analysis, we edit the *ModelSpecification* object to use linear models for the motion probabilities. Additionally, users can create a data class to specify a user-defined functional form. This is demonstrated in the first sample analysis, where we create the sigmoidal decay and sigmoidal increase data classes to be used for the motion probabilities. This flexibility allows users to tailor their analyses to their specific research requirements.

**Table 1:** Standard function options for model fitting

Function name	Functional form	Parameter signs	Uses
Exponential Decay	$y=a{e}^{bx}+c$	a > 0 b < 0 c > 0	Measures that decrease over time/coverage, approaching an asymptote; Default option for activity, P_++_, and P_+0_
Asymptotic Increase	$y=a({e}^{bx}-1)$	a < 0 b < 0	Measures that start at or near zero and increase over time/coverage, approaching an asymptote; Default option for coverage, P_+-_, P_+0_, and P_00_
Linear Decrease	*y=ax+b*	a < 0 b > 0	Measures that decrease linearly over time/coverage; Sometimes used for P_++_, and P_+0_
Linear Increase	*y=ax+b*	a > 0 b > 0	Measures that increase linearly over time/coverage; Sometimes used for P_+-_, P_+0_, and P_00_
Sigmoidal Decay	$y=\frac{a}{1+{e}^{-b(x+c)}}$	a > 0 b < 0 c < 0	Measures that begin and remain at a high or saturated level before decreasing over time/coverage approaching a lower asymptote; Sometimes used for P_++_, and P_+0_
Sigmoidal Increase	$y=\frac{a}{1+{e}^{-b(x+c)}}$	a > 0 b > 0 c < 0	Measures that begin at a low level before increasing over time/coverage approaching a higher or saturated asymptote; Sometimes used for P_+-_, P_+0_, and P_00_

While maintaining flexibility in function specification, the default functions embedded in *opynfield*’s model fitting module, as well as those used in the sample analyses, boast high interpretability. Taking the time versus activity relationship as an example, parameters *a*, *b*, and *c* individually influence distinct aspects of exploration. Differences in these parameter values offer insights into the underlying processes of initial activity/neophilia (parameter a), habituation speed (parameter b), and steady-state activity levels (parameter c), respectively.

### Module 6: Statistical Tests

In order to leverage the interpretability of the model parameters, the next module (*stat_test*) performs statistical tests directly on the individuals’ best-fit parameters. The primary statistical tool used in *opynfield* is the Multiple Analysis of Variance (MANOVA) test. In this test, each parameter derived from the model fits assumes the role of a dependent variable, while the experimental groups act as the independent variable. This approach allows for a holistic assessment of group-level variations in behavior. Given the substantial number of tests conducted, executing a full MANOVA model demands considerable data. Thus, *opynfield* also introduces the concept of "sub-tests." These sub-tests are MANOVA analyses on all parameters associated with a specific relationship, offering a pragmatic alternative for scenarios with limited data availability. By default, *opynfield* fits models for all versions of the motion probabilities and models each variable by time as well as by each version of coverage. Thus, sub-tests are also useful when you are only interested in a subset of these relationships, such as using only the “given previous” versions of the motion probabilities and using only percent coverage as an independent variable.

Following a statistically significant MANOVA (or MANOVA sub-test), the Analysis of Variance (ANOVA) test is applied to each parameter that was included in the MANOVA. Here, the parameter functions as the dependent variable, and, once again, the experimental group constitutes the independent variable. The ANOVA tests pinpoint specific parameters that contribute significantly to observed differences.

In the final stage of the statistical evaluation, pairwise t-tests are conducted on each parameter. This identifies specific groups exhibiting significant differences from one another and provides a nuanced understanding of behavioral variation. The execution of all tests is automated through the *run_tests* function, streamlining analysis.

The outcome of the MANOVA, ANOVA, and t-tests is documented in tables saved to a text file. For determining the significance of each test, users are directed to examine the Pr > F, PR(> F), or pvalue-hs columns, respectively. Additionally, the coef column of the pairwise t-test results should be used in conjunction with the exported model parameters to determine effect sizes and biological significance. Comprehensive details regarding the statistical tests, their output, and interpretations can be found in the package documentation.

### Module 7: Plotting

The *opynfield* package culminates in the final module (*plotting*), which automates plot generation, allowing users to visualize the results derived from the preceding modules. The plotting module creates five distinct categories of plots, tailored to different stages of analysis.

First, the function *plot_traces* depicts the trajectory of the tracked organism alongside the inferred arena boundary. These plots may be useful early in the analysis to ensure accurate data reading, detect tracking errors, and identify anomalies in activity or arena boundary distortions.

Next, the function *plot_all_individuals* illustrates specific behavioral measure relationships for individual subjects. Customizable *PlotSettings* parameters can control the addition of model fit curves or equation layers. These plots can be used to validate the function chosen to model activity measures (e.g., determine if the activity exhibits an exponential decrease over time).

Additionally, the function *plot_all_solo_groups* reveals the average relationship between two variables for a specific group. Here, too, customizable *PlotSettings* parameters can control the inclusion of additional layers such as model fit curves, model equations, and SEM error bars on the average data points. This offers a collective view of the group dynamics.

The solo group plots are best utilized in conjunction with the group component plots generated by the function *plot_components_of_solo_groups*. These plots present the average relationship for a group alongside the raw data from all the individuals within that group. In addition to the solo group plot parameters, users can optionally specify the inclusion of individual model fits in the background using *PlotSettings*. These plots aid in choosing the optimal versions of coverage and the motion probabilities for a specific application and can verify that the group dynamics align with the individual-level trends.

Finally, the group comparison plots generated by *plot_all_group_comparisons* show the averages of all groups in the experiment. Users retain the same optional layers as in the solo group plots. These plots are used at the end of an experiment to visually highlight the differences between groups identified in the statistical test module.

Numerous *PlotSetting* parameters govern the generation and storage of plots. Detailed information on these parameters is available in the tutorial and documentation.

### Availability

The *opynfield* package is a powerful tool for analyzing open field exploration tracking data and is readily accessible to researchers. The software is freely available on the Python Packaging Index (PyPI) or can be obtained directly from its GitHub repository (https://github.com/EllenMcMullen/opynfield).

The GitHub repository serves as a comprehensive hub, hosting the software package alongside resources to facilitate use. In addition to documentation, a tutorial is provided as a Jupyter notebook within the repository, allowing users to run analyses with the included test datasets. The tutorial guides users through core functions, enabling them to practice with curated data before adapting the workflow to their own datasets. The repository also features a user-support community, including a discussion board where users can pose questions to other users or the authors. This community resource assists both individual users and the broader group by addressing shared challenges and questions. It also serves as a conduit for feature requests, allowing users to suggest additions or modifications to help ensure that opynfield evolves in line with user needs.

The package utilizes several other open-source packages, including pandas (The Pandas Development Team, 2023), scipy (Virtanen et al., 2020), numba (Lam et al., 2015), statsmodels (Seabold & Perktold, 2010), and matplotlib (Hunter, 2007). Full dependency information can be found in the supplementary materials (Online Resource 1). opynfield is available under the GPL-3.0 license. The version used in this paper is archived on Zenodo (https://doi.org/10.5281/zenodo.15794680). For further information on installation and usage, see Online Resource 2.

## Results and Discussion

### Overview

Here, we demonstrate three important characteristics of *opynfield* analysis, underscoring advancements over previous open field exploration analysis methods. First, the multi-dimensional and time-dense data produced can pose challenges for traditional analytical techniques, leading to an inflated false positive rate, whereas the statistical approach in *opynfield* does not. Second, the method employed to summarize data from experimental groups proves instrumental in validating coverage as a measure of learning. Finally, the parameter-based statistical analysis is capable of discerning distinctions in exploratory patterns and identifying the specific components of exploration that diverge between experimental groups, hinting at the physiological processes involved. In addition to these features that we demonstrate using *Drosophila* data, we also include an analysis of *Mus musculus* open field data to demonstrate the cross-species utility of *opynfield*, show how certain analysis parameters can be customized based on the subject’s exploratory patterns, and demonstrate the biological insights that *opynfield* analyses can detect.

While many solutions exist to track animal position, whether in real-time or derived from pre-recorded video, there has been a lack of software dedicated to the analysis of tracking data until now. This void necessitates the creation of custom scripts for each experiment or experimenter, resulting in decreased replicability and impeding comparison across experiments. Additionally, it presented a barrier to the adoption of more intricate behavioral measures, such as coverage. Now *opynfield* fills this gap, empowering users to analyze open field exploration data and to go beyond simple minute activity bins, producing high-density models of multiple exploratory measures with integrated statistical tests that isolate specific components of exploratory changes.

All flies used in these validation studies were 2–5-day-old males raised on yeast-cornmeal agar food at 25 °C on a 12-h light/dark cycle. The Canton-S stocks were obtained from the Laboratory of Ronald Davis (Scripps FL). Blind flies (*w*^*1118*^) were also obtained from the Davis Lab and backcrossed into the Canton-S background. All tracks were recorded in clear plexiglass arenas with a diameter of 8.4 cm using the BuriTrack program, unless otherwise specified. A single fly was aspirated into the arena through a hole in the arena lid, then the lid was positioned with the hole outside of the arena walls so that the fly could not exit. Arenas were illuminated with fluorescent bulbs and positioned so that each arena received equal light intensity. Tracking was performed at room temperature, approximately 23 °C, and lasted 10 min with a sampling rate of 30 Hz.

The care and handling of mice in this study were conducted in strict accordance with the Guide for the Care and Use of Laboratory Animals (NRC, 8th edition). All experimental protocols were approved by the Institutional Animal Care and Use Committee at the University of Texas Health Science Center at San Antonio. Male and female C57BL/6J mice (Jackson Laboratory, Bar Harbor, ME; stock #000664) were used as wildtype controls in behavioral experiments at three age groups: young (4–5 months), middle-aged (9–12 months), and old (22–30 months). Male and female KMO-/- mice were generated as described in Parrott et al. (2016) and tested at the same age groups. Mice were housed in standard cages in groups, with ad libitum access to food and water, and maintained on a reverse 12-h light/dark cycle. Their general health was monitored daily by veterinary technicians or research staff.

Mouse tracking was performed using ANY-maze 7.3 (Stoelting, Wood Dale, IL), with video recorded by a C920 HD camera at 30 fps (Logitech, San Jose, CA). A single camera was centered 2 m above the arena to simultaneously capture all four compartments of the open field apparatus. The custom-built open field apparatus consisted of four individual compartments, each measuring 40 cm × 40 cm × 40 cm, with cylindrical inserts (38 cm in diameter, 30 cm in height) to create four separate circular open fields. The open field apparatus was constructed from acrylic plastic. ANY-maze software was configured to treat each circular open field as an independent apparatus for data collection. Each mouse was tracked for 10 min. All testing was conducted in a noise-controlled room, at a temperature of 21 °C, with an illumination level of 600 lx.

### Characteristic 1: Acceptable False Positive Rate

The primary goal of *opynfield* is to identify whether the experimental groups differ in specific exploratory behaviors. For example, we may want to know whether one group has lower activity at a given percent coverage, indicating faster habituation. However, comparing arbitrary curves is a difficult problem and has the potential to underestimate variance, resulting in inflated false positive rates. To overcome this, we adopted a novel approach of parameterizing these relationships, enabling a direct comparison of individual model parameters. This not only significantly reduces dimensionality but also enhances interpretability.

To validate the efficacy of this approach, we conducted an experiment involving 110 Canton-S flies from a single population recorded in the open field. The tracks were randomly divided into two equal-sized groups, CS1 and CS2. Given that these tracks originated from the same population, any significant differences in the examined relationships between CS1 and CS2 would signal a potential error in our method. Out of 35 parameters of interest, only two yielded statistical significance in both the MANOVA sub-test and the parameter ANOVA (Table 2). This outcome matches our selected type I error rate (2/35 = 0.057 ≈ 0.05), underscoring the reliability of our approach. While additional parameters showed low *P*-values in the ANOVA results, these did not follow a statistically significant MANOVA sub-test and thus are ignored. Likewise, when interpreting other results, we only consider a parameter effect to be significant when it follows a significant sub-test result.

**Table 2:**
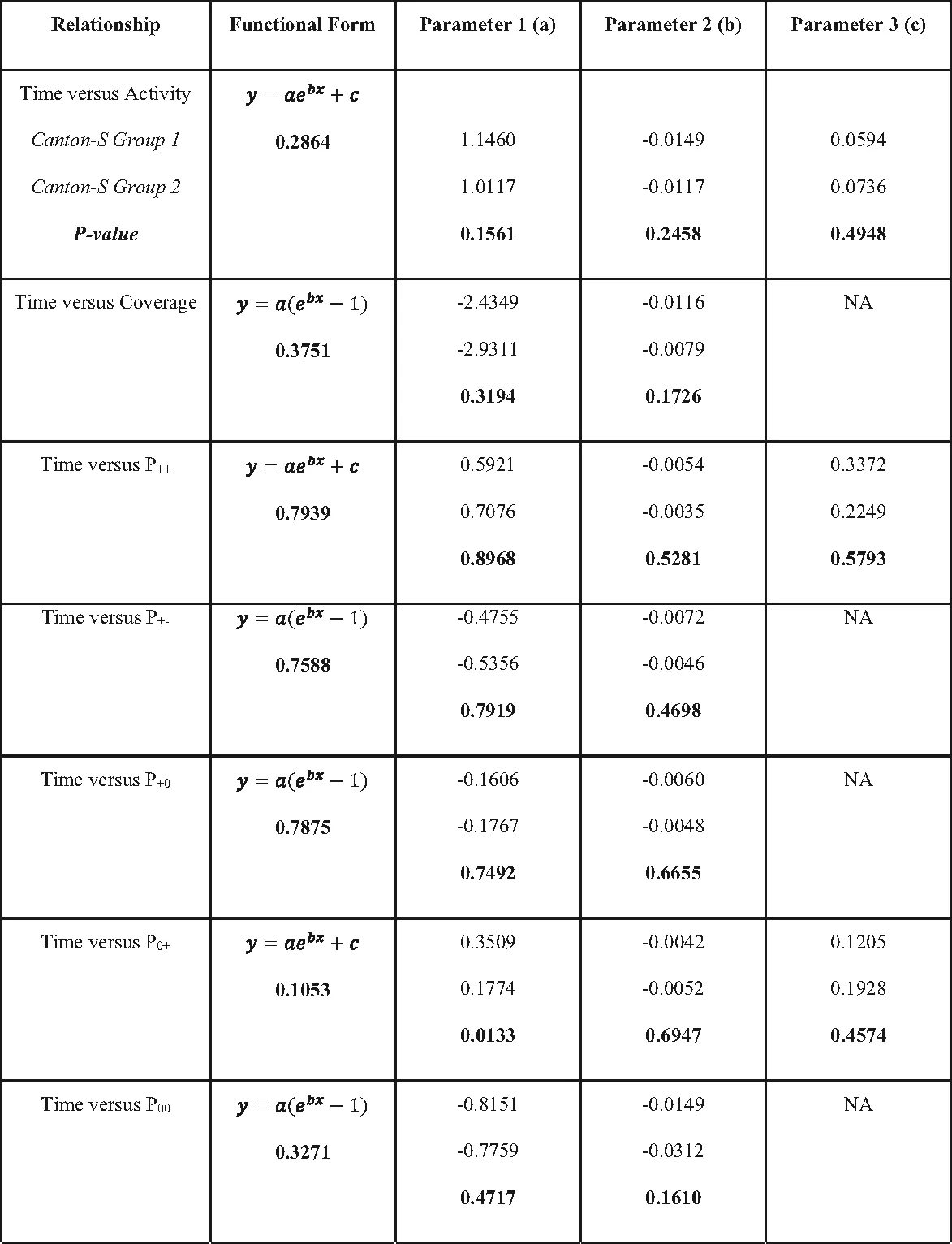

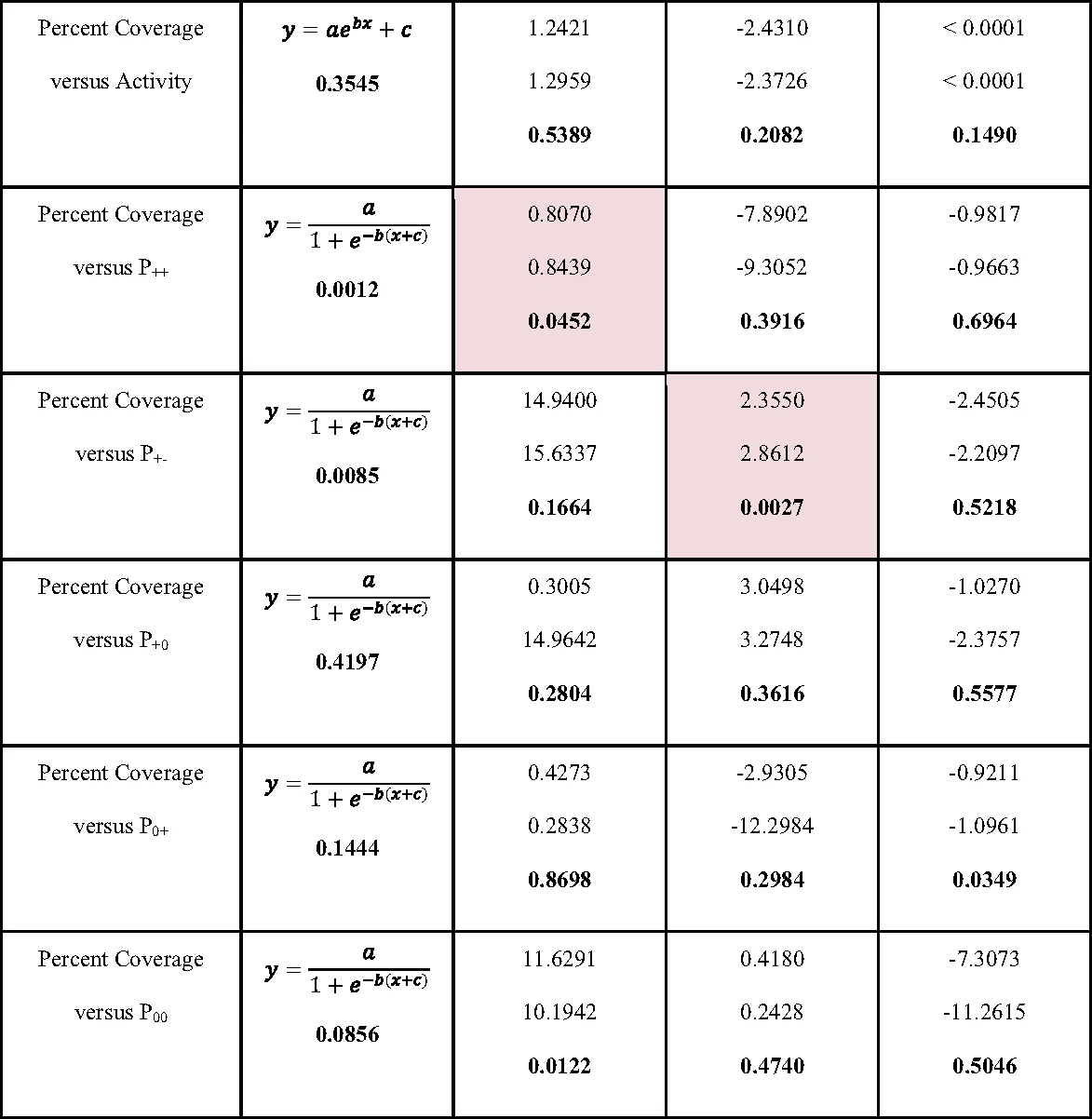
Comparison of Canton-S groups yields an acceptable false positive rate

Model parameters from Canton-S flies. Cells provide the parameter from Group 1, the parameter from Group 2, and the *P*-value from the t-test between them. NA is reported in the Parameter 3 column when a relationship is modeled with only two parameters. The functional form of the model used to fit the parameters is provided for each relationship. The *P*-value for the MANOVA subtest on that row of parameters is provided under the functional form. Red cell shading indicates statistical significance in both the MANOVA sub-test and the parameter ANOVA (*P*-value < 0.05)

**Fig. 3:**
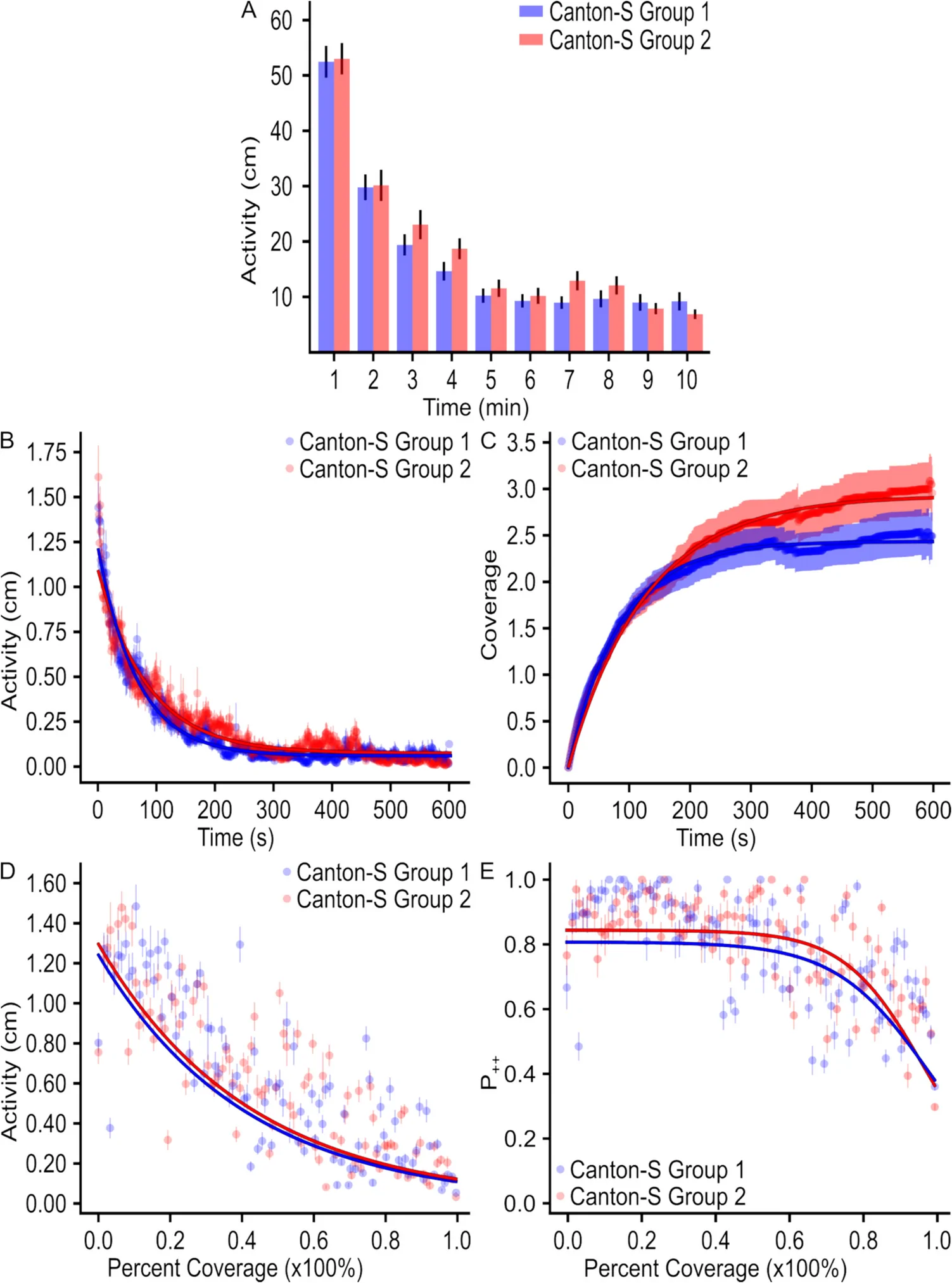
Comparison of Canton-S groups using *opynfield* provides more information than traditional minute-bin-based analysis. (**a**) The time versus activity relationship reported in minute bins provides coarse insights into activity differences between groups. The continuous time versus activity relationship, as reported from *opynfield* (**b**), provides a closer look at the experiment’s habituation rate. The *opynfield* package additionally provides insight into learning as a measure of coverage (**c**). Additionally, *opynfield* allows for analysis of activity (**d**), P_++_ (**e**), and other variables as a function of percent coverage. Colored bars in A represent mean activity levels within each one-minute time bin. Black error bars represent the standard error of the mean (SEM). In B-E, points represent mean values within each bin, error bars represent SEM, and solid curves represent the model fits. The model forms used include an exponential decrease model in B and D, an asymptotic increase model in C, and a sigmoidal decay model in E. Note that bins contain a variable number of points and the models are fit on the underlying data, which explains the appearance of more points being present above the model curves than below in some panels

Additionally, we utilized this dataset to compare *opynfield*’s results to prevalent methods. Standard practice involves binning activity into minute-long intervals, aiming to mitigate dimensionality for the subsequent application of ANOVA or t-tests at each time point. While these approaches can identify points of activity divergence (Fig. 3A), *opynfield* offers a more nuanced perspective by pinpointing the specific parameters responsible for these differences (Fig. 3B). This insight provides valuable hints about the underlying mechanisms contributing to variations in activity.

Furthermore, *opynfield* is not restricted to the analysis of activity, and thus can facilitate deeper insights. For example, evaluating coverage over the course of the experiment (Fig. 3C) provides an estimate of the required number of learning trials for habituation. Similarly, examining the correlation between activity and percent coverage or P_++_ (Fig. 3D and E) delivers insights into the interplay of these variables. Thus, *opynfield* surpasses the limitations of current practices and offers a comprehensive toolkit for behavioral exploration analysis.

### Characteristic 2: Validated Coverage as a Measure of Learning

Beyond establishing an acceptable error rate, our investigation with *opynfield* sought to corroborate coverage as a reliable metric of learning. If habituation indeed involves learning the arena boundary, we hypothesized that, despite different levels of activity, flies should reach similar levels of coverage given enough time. Additionally, activity should correlate with the extent of learning achieved, quantified by visits to distinct boundary segments, irrespective of arena size. To test this hypothesis, we examined Canton-S behavior in arenas with diameters of 8.4 cm and 5.0 cm. Subjects in this experiment were tracked using Ethovision under the same experimental conditions as elsewhere.

**Fig. 4:**
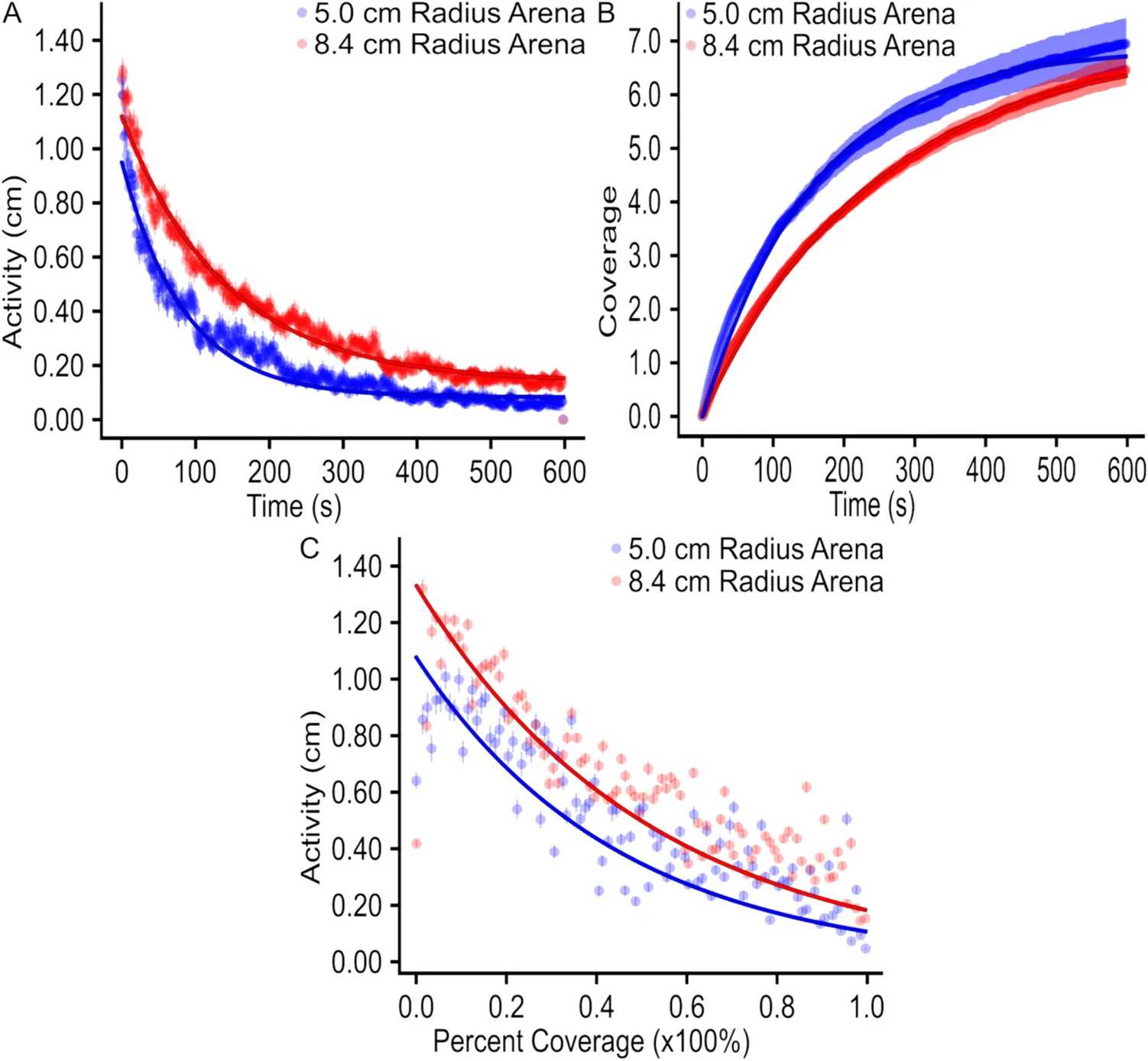
Despite higher activity levels, flies in larger arenas approach equivalent final coverage asymptotes. The plot of time versus activity (**a**) demonstrates that flies in 8.4 cm arenas exhibit increased activity. Despite this, they show a slower increase in coverage but approach an equivalent coverage asymptote (**b**). We also see that they have higher activity as a function of percent coverage (**c**), indicating that they must explore more for an equivalent increase in coverage. In all panels, points represent mean values within each bin, error bars represent SEM, and solid curves represent the model fits. The model forms used include an exponential decrease model in A and C, and an asymptotic increase model in B. Note that bins contain a variable number of points and the models are fit on the underlying data, which explains the appearance of more points being present above the model curves than below in some panels

As anticipated, flies in the larger arena exhibited heightened activity in the time domain (Fig. 4A), a logical outcome given the increased spatial area for the fly to navigate and learn. This relationship is also evident in the model parameter comparisons (Table 3). The *c* parameter, which represents the expected final activity level, is approximately 40% higher in the 8.4 cm arena flies (0.083 for 5.0 cm arena flies, 0.139 for 8.4 cm arena flies), as compared to an approximately 20% difference observed by chance between the Canton-S groups. The magnitude of the *b* parameter, which represents the rate of exponential decay in activity, is approximately 36% greater in the 5.0 cm arena flies (0.011 for 5.0 cm arena flies, 0.007 for 8.4 cm arena flies), as compared to an approximately 30% difference by chance in the Canton-S groups. Thus, flies in the 8.4 cm arena start at the same activity level as those in 5.0 cm arenas but reduce their activity level more slowly and maintain a higher final activity level.

**Table 3:**
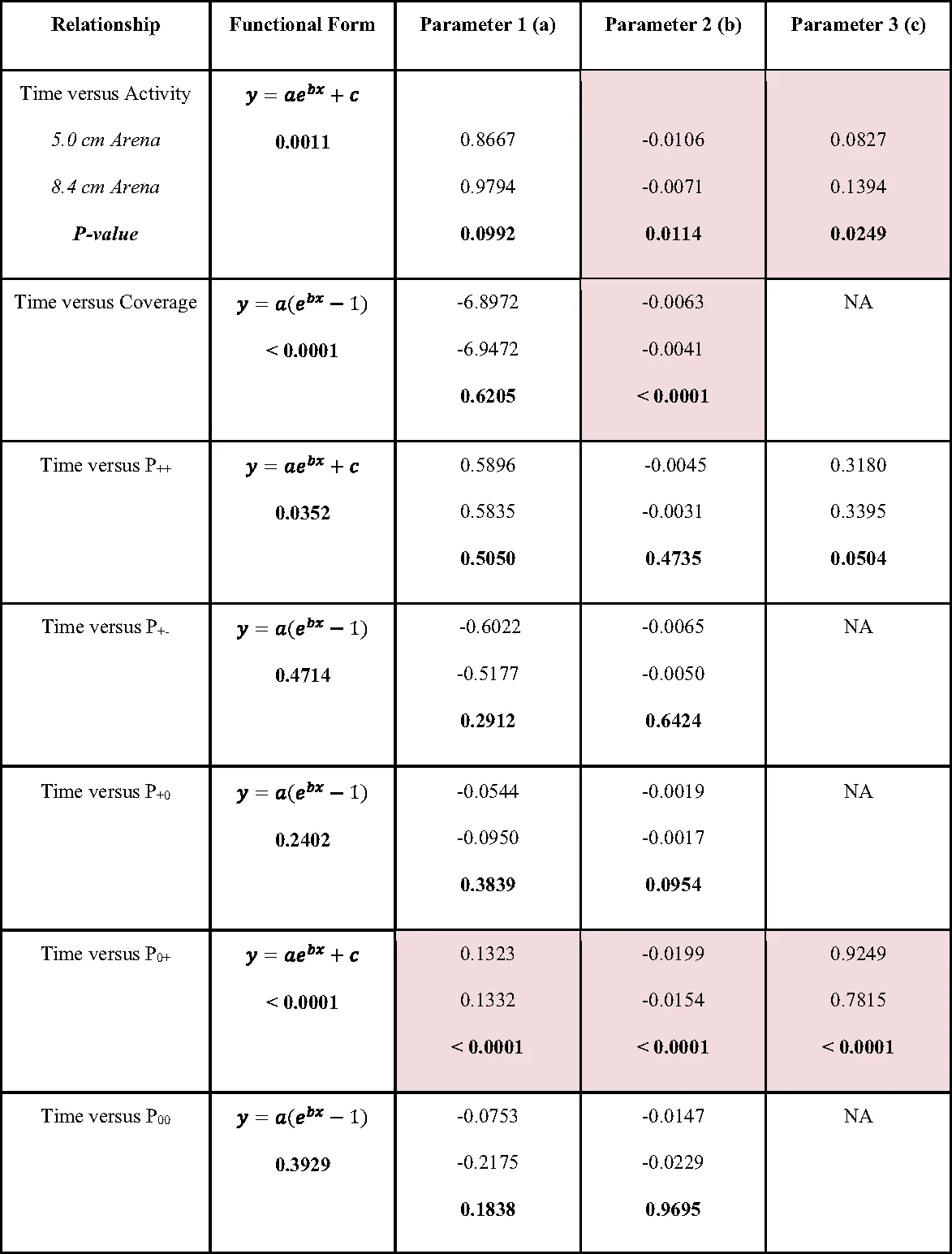

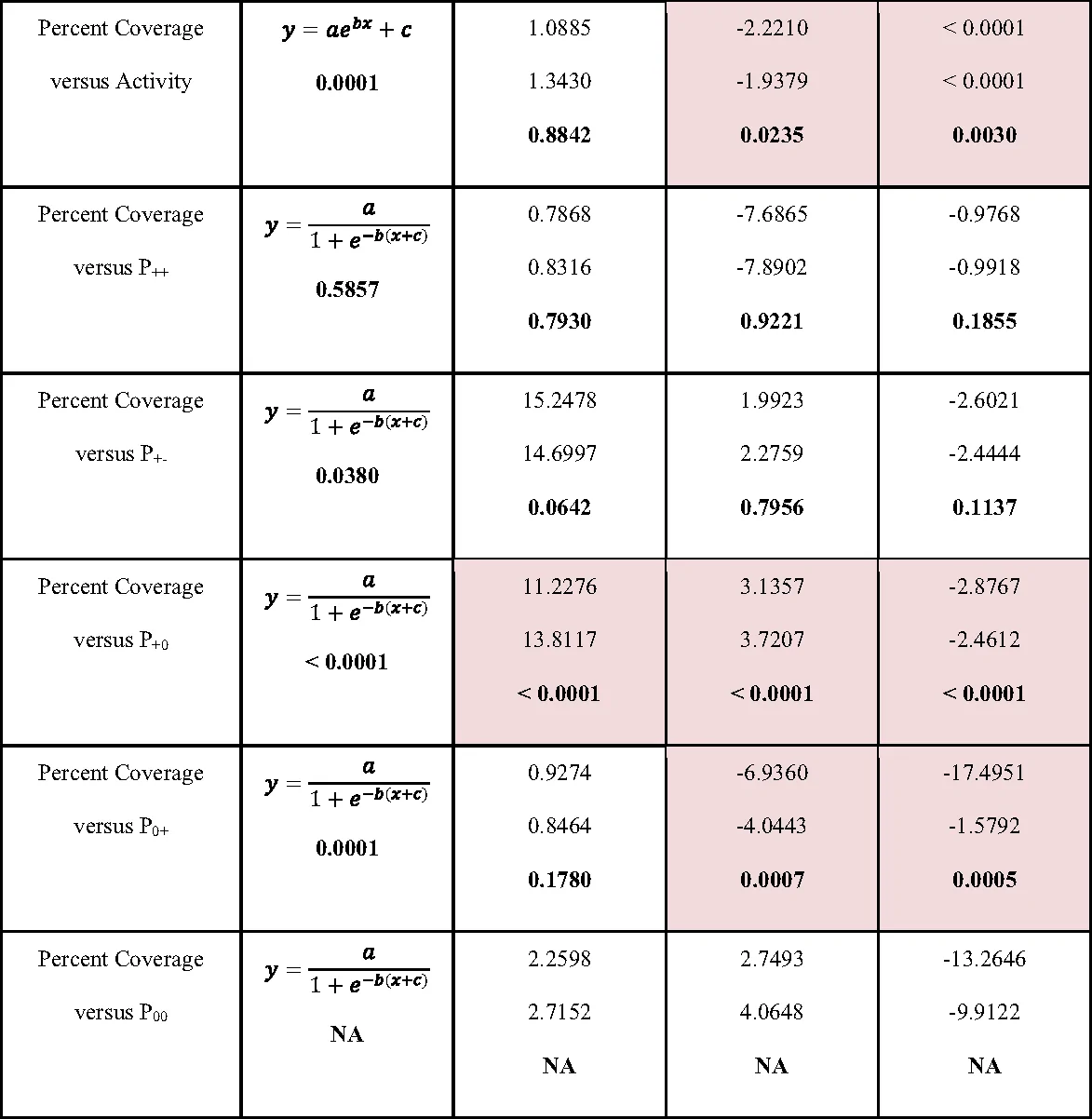
Analysis of coverage demonstrates that flies in larger areas exhibit higher activity but approach the same number of learning trials

Model parameters from Canton-S flies. Each cell provides the parameter from flies in 5.0 cm diameter arenas, the parameter from flies in 8.4 cm diameter arenas, and the *P*-value from the t-test between them. NA is reported in the Parameter 3 column when a relationship is modeled with only two parameters. The functional form of the model used to fit the parameters is provided for each relationship. The *P*-value for the MANOVA subtest on that row of parameters is provided under the functional form. Red cell shading indicates statistical significance in both the MANOVA sub-test and the parameter ANOVA (*P*-value < 0.05). NA is reported in place of the *P*-value when there was too little data to conduct a test

By examining the time versus coverage relationship (Fig. 4B), we see that they are approaching equivalent total coverage values, also evidenced by the model parameters in Table 3. The *a* parameter denotes the asymptote value of the time versus coverage relationship, and both groups approach approximately 7 visits to each area boundary segment. The magnitude of the *b* parameter, which represents the rate at which the increase in coverage changes, is approximately 35% greater in the 5.0 cm arena group (0.00631 for 5.0 cm arena flies, 0.00410 for 8.4 cm arena flies), as compared to a difference of 33% observed by chance in Canton-S flies. This means the flies in 8.4 cm arenas require more time to complete the 7 visits. Despite the equivalent coverage required for full learning, flies in larger arenas exhibit a slower rate of coverage increase.

Finally, we can examine the activity versus percent coverage relationship. Here, while the t-test revealed a statistically significant difference in the *c* parameter, the values of *c* are essentially zero, meaning both groups are expected to cease activity after completing sufficient learning trials. On the other hand, the magnitude of the *b* parameter is about 13% greater in the 5.0 cm group (2.22 for 5.0 cm, 1.94 for 8.4 cm), as compared to a 2% difference between the Canton-S groups by chance. This means that flies in the 8.4 cm arenas decrease their activity at a slower rate, even as a function of the learning achieved within the experiment. Accordingly, when we examine the percent coverage versus activity relationship, we can see that the groups remain distinct, with flies in the large arenas exhibiting higher activity at equivalent proportional learning (Fig. 4C).

Taken together, these results suggest that higher-order processes may be involved. Despite having higher overall activity, flies in large arenas reach the coverage level required for full learning more slowly. Thus, they may exhibit increased activity at a given level of coverage to compensate for the larger distance they need to traverse to revisit each arena boundary segment for a new learning trial (and thereby increase their coverage). Further work should be done using additional arena sizes to determine if this gap in activity persists in smaller arenas. If the effect is only present in the largest arenas, it may indicate not a difference in exploratory drive, but rather a physiological limit on their ability to maintain the activity level required to visit and learn each arena boundary segment at the expected habituation rate.

### Characteristic 3: Distinguishes Between Different Types of Activity Changes

In addition to establishing an acceptable false positive rate and validating coverage as a metric of learning, *opynfield* can be used to delineate the specific mechanisms underlying exploratory differences between experimental groups. To illustrate this, we examined the exploratory behaviors of mutant *white*^*1118*^ flies in contrast to their wildtype counterparts, Canton-S flies.

**Fig. 5:**
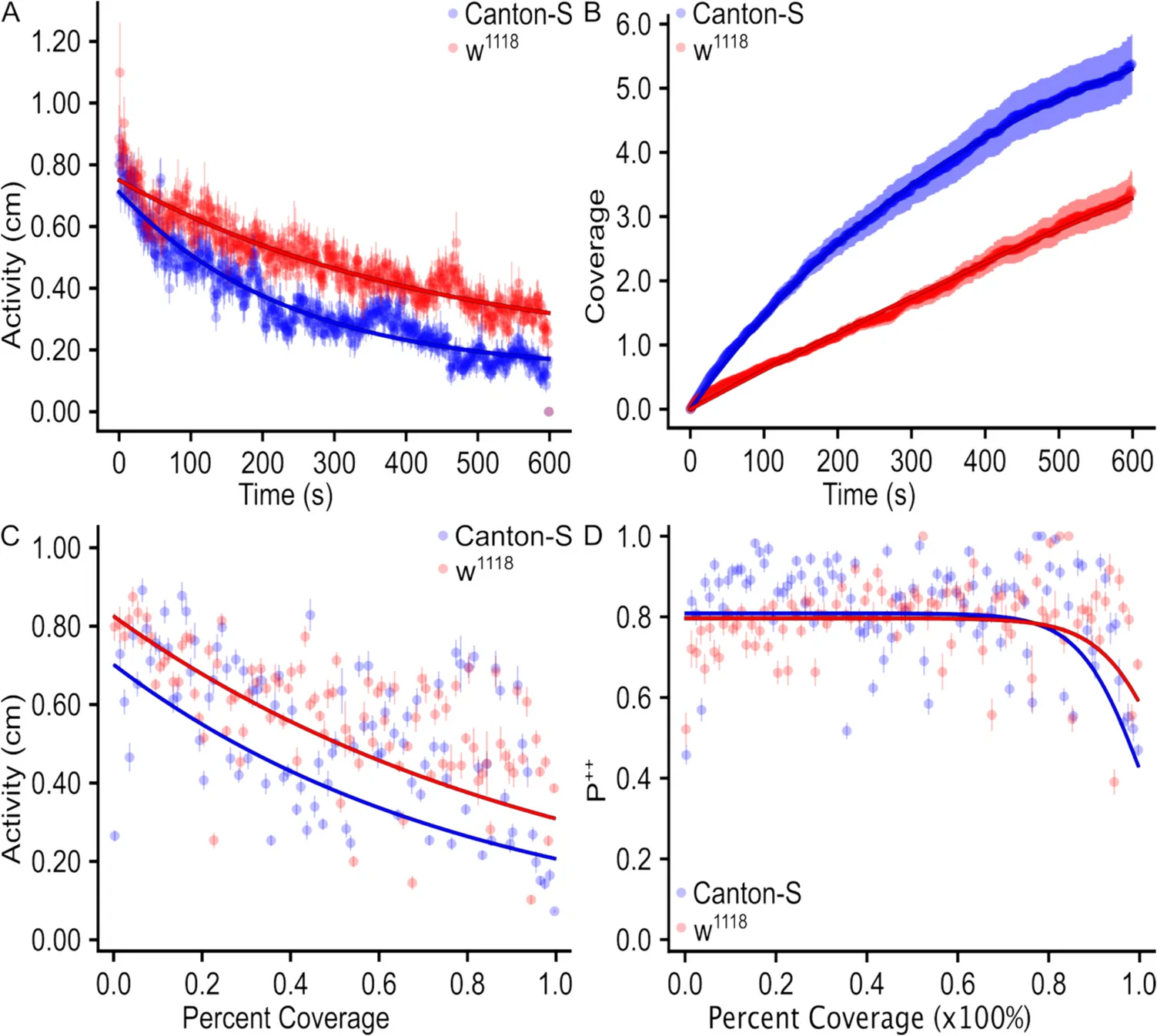
Visually impaired flies exhibit learning deficits rather than hyperactivity. The plot of time versus activity (**a**) shows that *white*^*1118*^ flies demonstrate similar initial activity to wildtype Canton-S levels but maintain higher activity levels for longer. Despite higher activity, they show decreased coverage (**b**). Unsurprisingly, they have higher activity as a function of coverage (**c**), given that they cannot learn from their visits. This is also demonstrated in the pattern of P_++_ (**d**), where *white*^*1118*^ flies maintain their directional persistence more than Canton-S flies. In all panels, points represent mean values within each bin, error bars represent SEM, and solid curves represent the model fits. The model forms used include an exponential decrease model in A and C, an asymptotic increase model in B, and a sigmoidal decay model in D. Note that bins contain a variable number of points and the models are fit on the underlying data, which explains the appearance of more points being present above the model curves than below in some panels

Mutant *w*^*1118*^ files, which have reduced visual acuity, typically exhibit increased activity and decreased habituation compared to wildtype flies, as they are less able to learn the arena boundary (Soibam et al., 2012a). This divergence is evident in the temporal activity plot (Fig. 5A) and in the statistical results comparing the model parameters (Table 4). The time-activity relationship was fit to an exponential decay model (Eq. 12), with parameters *a*, *b*, and *c*. Parameter *a*, reflecting the initial activity level, exhibited no statistically significant difference between the groups, indicating that the groups had equal neophilic drive to explore. Parameter *b*, representing the rate of activity decrease, was 46% smaller in the *w*^*1118*^ group, suggesting a slower habituation rate compared to the 22% difference between Canton-S groups occurring by chance. Additionally, parameter *c*, the steady-state activity level, was 37% higher in the *w*^*1118*^ group, indicating a sustained elevated activity level in the absence of visual input, compared to the 23% difference between Canton-S groups. Despite the apparent increase in overall activity, the *w*^*1118*^ group demonstrated reduced coverage over the 10-min experiment duration (Fig. 5B). This phenomenon could be primarily attributed to decreased edge-dwelling behavior among *w*^*1118*^ flies.

**Table 4:**
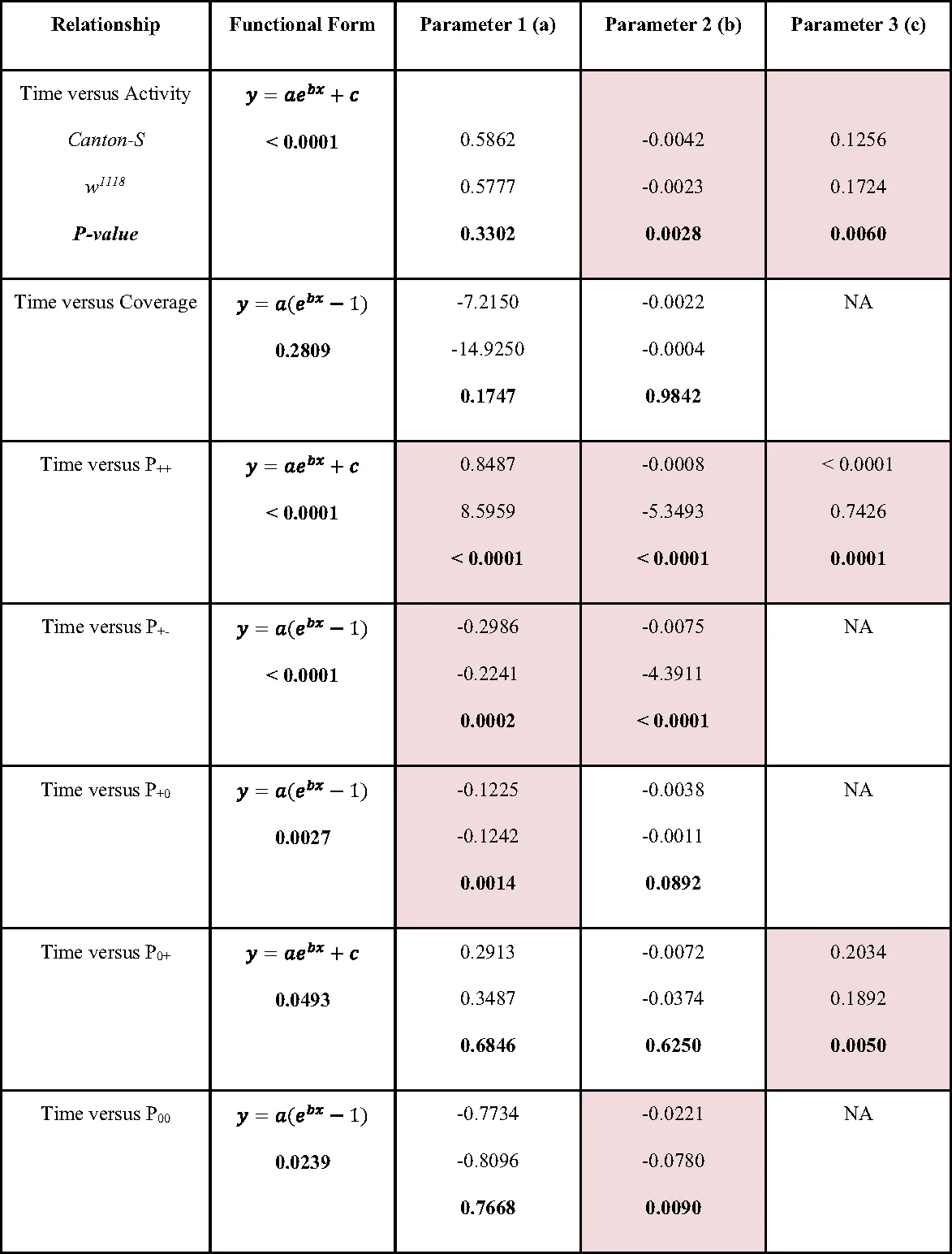

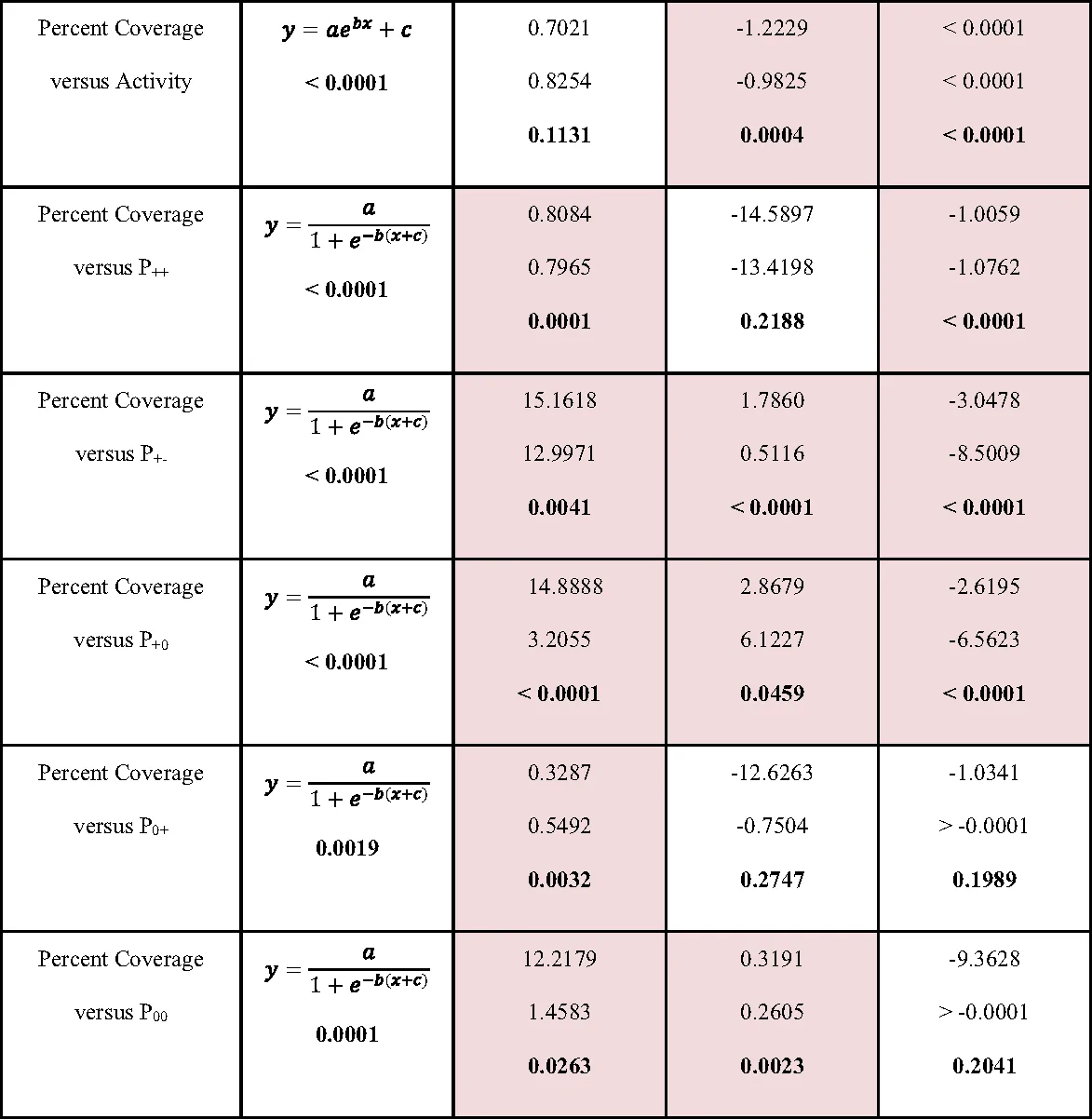
Analysis with *opynfield* distinguishes between kinds of exploration deficits

Model parameters from Canton-S and *w*^*1118*^ flies. Each cell provides the parameter from the Canton-S group, the parameter from the *w*^*1118*^ group, and the *P*-value from the t-test between them. NA is reported in the Parameter 3 column when a relationship is modeled with only two parameters. The functional form of the model used to fit the parameters is provided for each relationship. The *P*-value for the MANOVA subtest on that row of parameters is provided under the functional form. Red cell shading indicates statistical significance in both the MANOVA sub-test and the parameter ANOVA (*P*-value < 0.05)

The results from analysis in the percent coverage domain further corroborated these trends and show amplified activity in visually impaired flies (Fig. 5C) due, presumably, to their slower rate of habituation (parameter *b*) and a higher sustained activity level (parameter *c*). This finding underscores that the augmented activity in *white* flies is not solely a result of fewer visits to the edge, leading to reduced learning opportunities. Instead, even with an equivalent number of edge visits, blind flies persist at elevated activity levels, highlighting their inherent inability to learn from the environment. This observation is further substantiated by the elevated P_++_ values across percent coverage (Fig. 5D), providing a comprehensive understanding of the exploration dynamics in *white* flies. These observations highlight the role of visual cues in habituation.

### Mouse Open Field Behavior

The previous results demonstrated the utility of *opynfield* in analyzing exploration data from *Drosophila*, but the novel open field test is also highly used to analyze behaviors in rodents. Thus, we tested *opynfield*’s ability to identify genotypic and age-class differences in *Mus musculus* open field exploratory behavior.

Age-related chronic inflammation shifts the kynurenine pathway toward oxidative and neurotoxic metabolism. Kynurenine 3-monooxygenase (KMO), a key rate-limiting enzyme in this pathway, is upregulated in response to age-related pro-inflammatory signals. Our work shows that KMO deletion reduces levels of oxidative and neurotoxic metabolites such as 3-hydroxykynurenine and quinolinic acid (Parrott et al., 2016; Garrison et al., 2018), and that it preserves both olfactory habituation (de la Flor et al., 2025) and spatial learning and memory in aging and old KMO-/- mice compared to wildtype controls (data unpublished). Based on these findings, we hypothesized that KMO deletion would also influence exploratory behavior and habituation in the open field. In this experiment, we compare wildtype (C57BL/6J) and KMO mutant (KMO-/-) mice at three ages: young mice (4–5 months), middle-aged mice (9–12 months), and old mice (22–30 months), in circular arenas.

Due to the two-factor design of this experiment, we also demonstrate the export of parameters from *opynfield* to perform additional statistical tests not yet included in *opynfield*. Briefly, a two-way MANOVA was performed on all specified model parameters. Then, a two-way ANOVA was performed on each model parameter separately to identify age effects, genotype effects, and interactions. Finally, a Tukey’s Honest Significant Difference test was performed to identify the specific age classes that differed from one another, and to identify the direction and magnitude of inter-group differences. The full supplemental analysis (Online Resource 3) was performed in R (Version 4.2.1) with packages stringr (Version 1.5.0) and multcomp (Version 1.4–25).

**Fig. 6:**
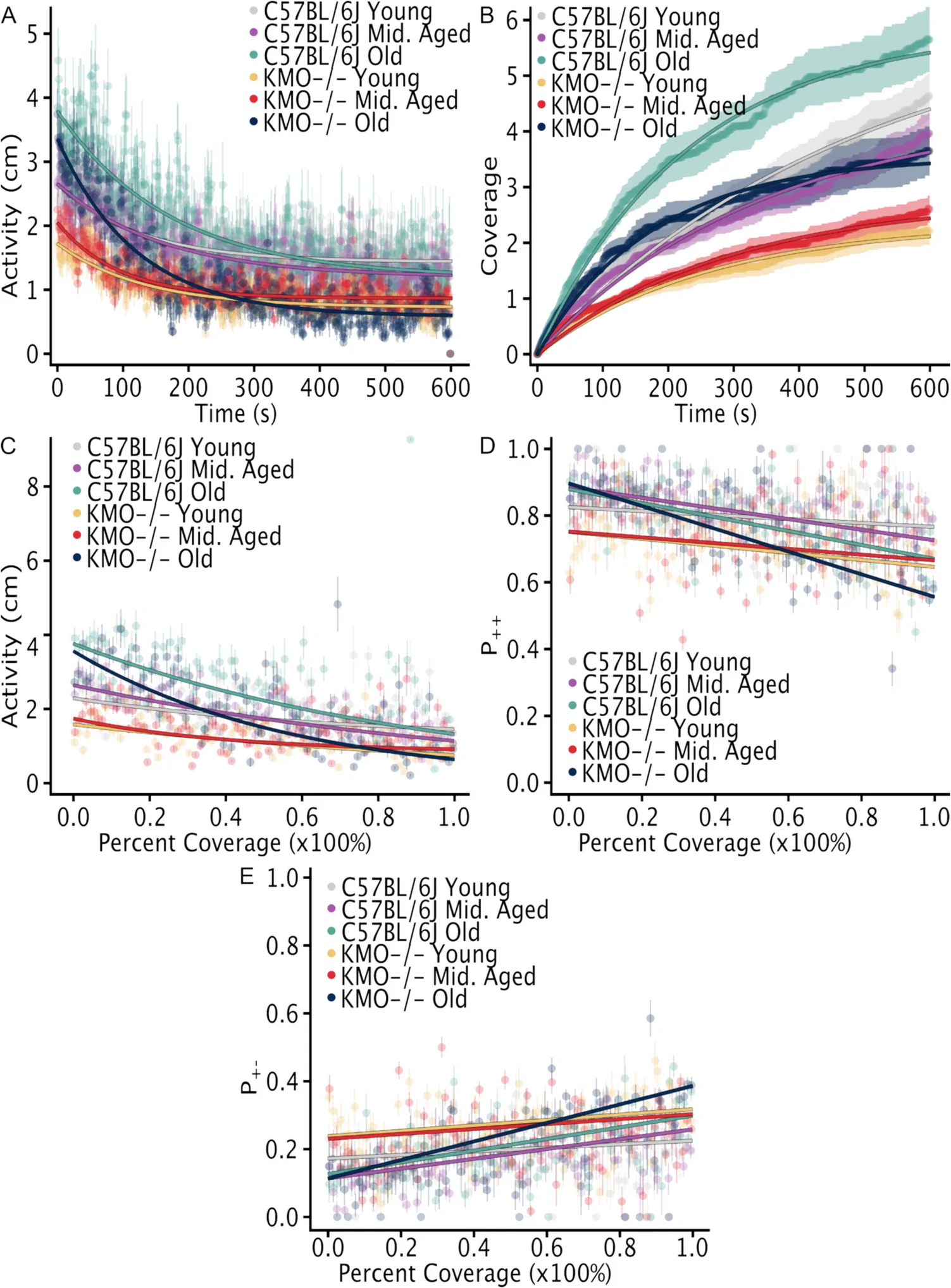
Genotype and age interact to shape exploratory patterns in mice. The plot of time versus activity (**a**) shows that wildtype mice and older mice exhibit higher activity than KMO-/- and younger mice. These different activity patterns lead to a slower accumulation of coverage over time (**b**) and a higher final coverage level in wildtype mice compared to KMO-/- mice. When examining activity as a function of percent coverage (**c**), the effects of main effect of KMO-/- mice having lower activity and lower coverage mostly cancel out, though they retain lower final activity than wildtype mice. However, age effects are present with older mice having higher initial activity, slower decreases in activity, and lower final activity than younger mice, though the slower habituation rate in old mice is not seen in the KMO-/- genotype. Wildtype mice exhibit higher P_++_ and slower decreases in P_++_ than KMO-/- mice (**d**), consistent with the activity patterns, while old KMO-/- mice exhibit higher P_++_ and faster decreases in P_++_ than younger mice. As P_++_ decreases, it is primarily P_+-_ that increases (**e**), with wildtype mice starting with lower P_+-_ and exhibiting a slower increase in P_+-_ than KMO-/- mice, while old KMO-/- mice exhibit lower initial P_+-_ and faster increases in P_+-_ than younger mice. In all panels, points represent mean values within each bin, error bars represent SEM, and solid curves represent the model fits. The model forms used include an exponential decrease model in A and C, an asymptotic increase model in B, a linear decrease model in D, and a linear increase model in E. Note that bins contain a variable number of points and the models are fit on the underlying data, which explains the appearance of more points being present above the model curves than below in some panels

There is an effect of both genotype and age on activity (Fig. 6A), with wildtype mice exhibiting higher initial activity than KMO-/- mice (P-value = 0.0439), and old mice exhibiting higher initial activity than middle-aged (*P*-value < 0.0001) or young (*P*-value < 0.0001) mice (parameter *a*, Table 5). Likewise, wildtype mice end with higher activity than KMO-/- mice (*P*-value < 0.0001), whereas young mice end with higher activity than middle-aged (*P*-value = 0.0191) or old (*P*-value = 0.0003) mice (parameter *c*). Note that final activity level comparisons are based upon the activity level asymptote (parameter *c*), rather than the activity level apparent in the graphs at t = 600 s. While we found both genotype-effects and age-effects in initial and final activity levels, the rate at which activity decreases (parameter *b*) is the same across genotypes, exhibiting only an age-effect; young mice decrease their activity faster than middle-aged (*P*-value < 0.0001) or old (*P*-value < 0.0001) mice.

**Table 5:**
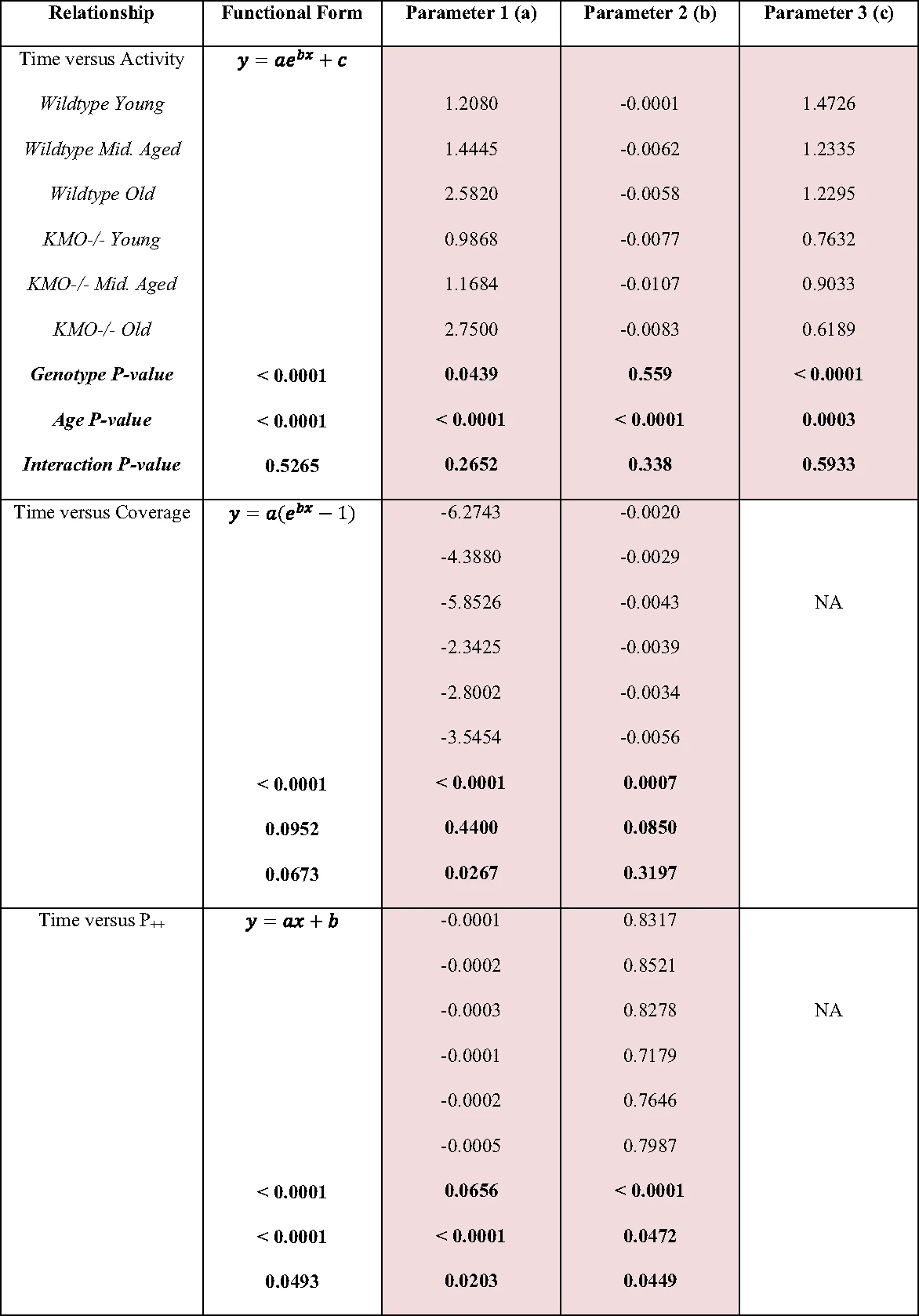

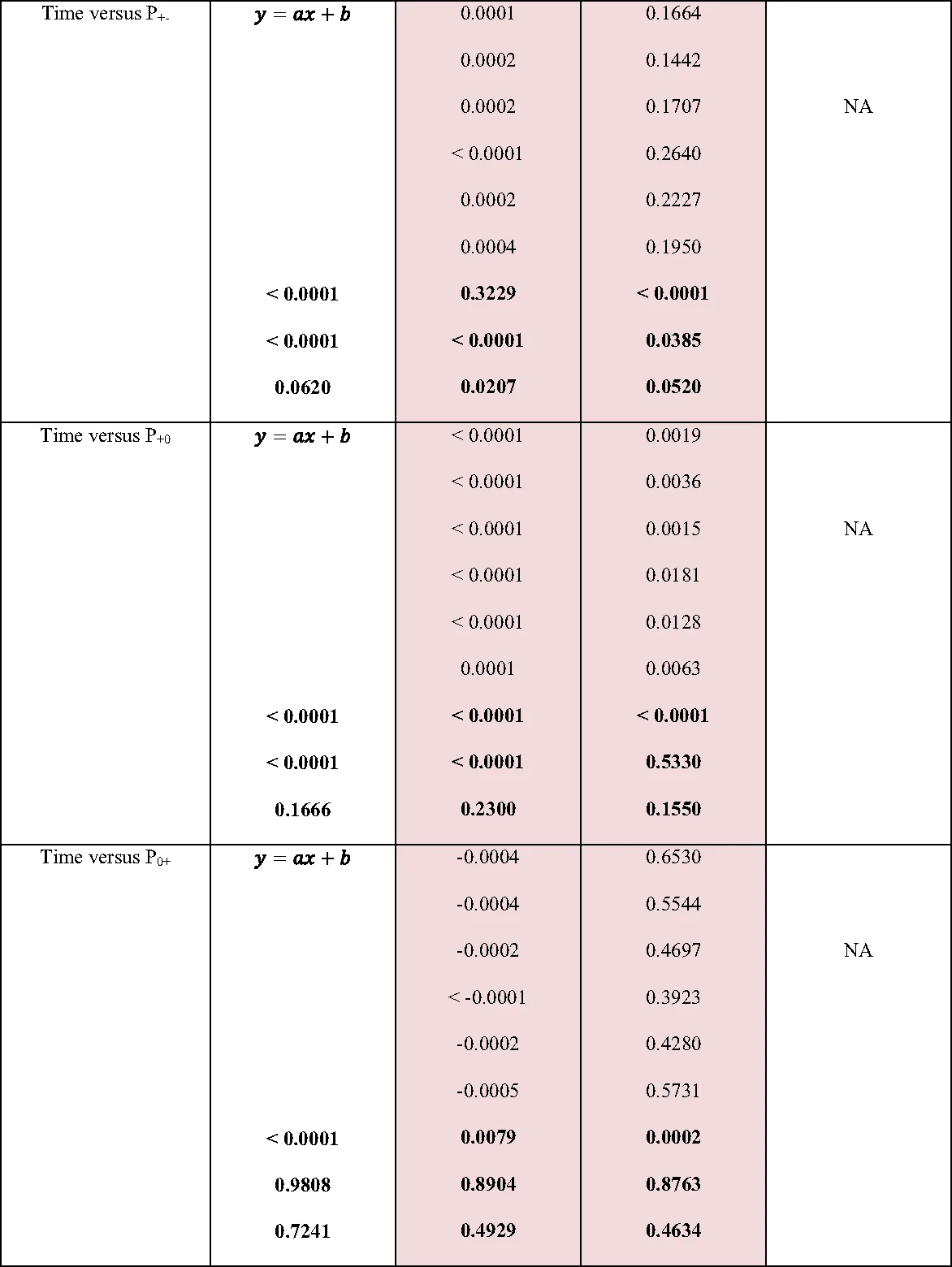

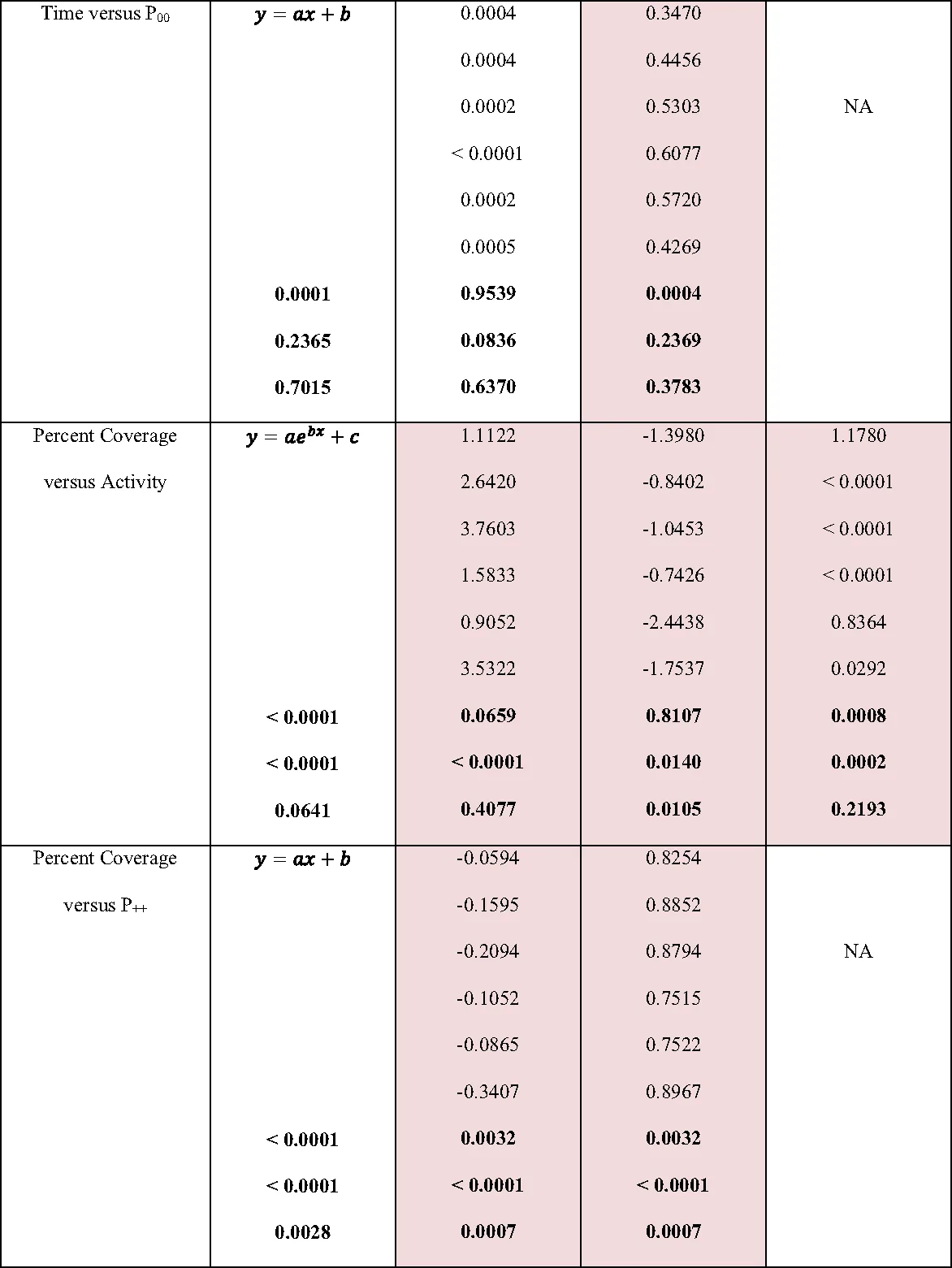

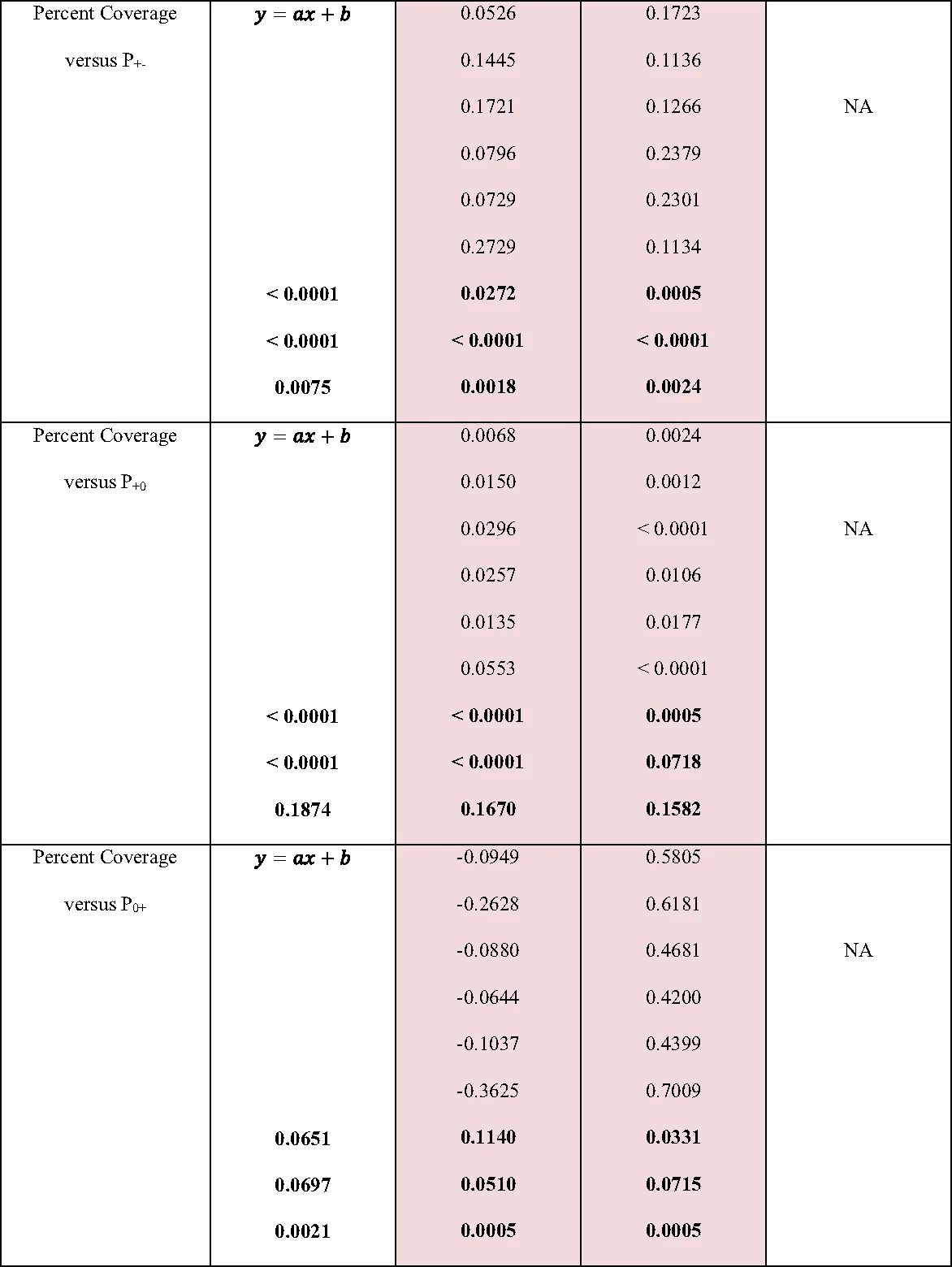

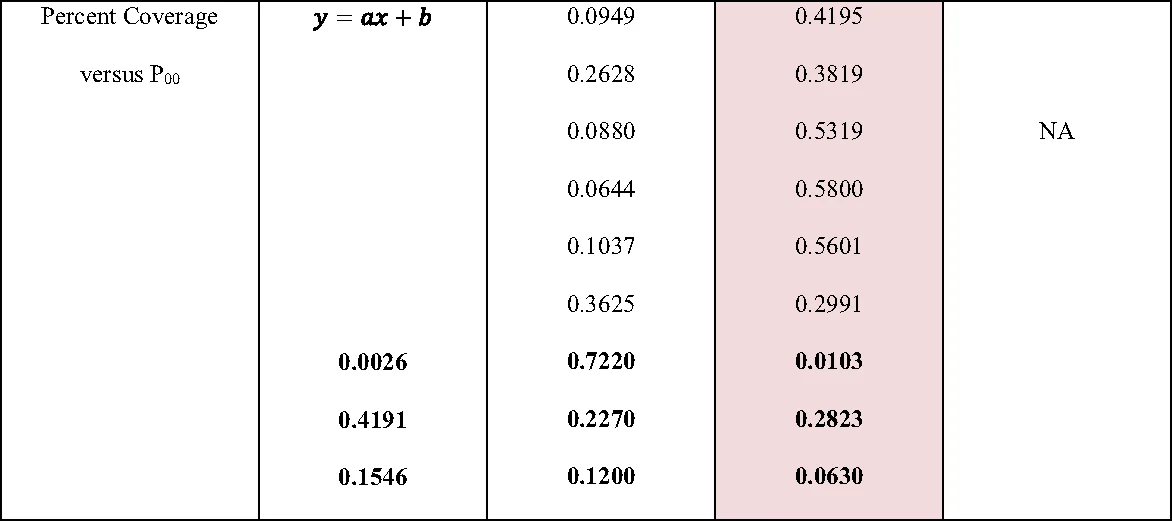
Analysis with *opynfield* identifies genotype and age differences in mouse exploration

Model parameters from wildtype and KMO-/- mice. Each cell provides the specified model parameter from the wildtype young group, wildtype middle-aged group, wildtype old group, KMO-/- young group, KMO-/- middle-aged group, and KMO-/- old group. Each cell also provides the *P*-values from the Two-Way ANOVA test on the specified parameter, including the genotype and age main effects as well as the interaction of genotype x age. Red cell shading indicates statistical significance in at least one of the MANOVA sub-test effects and in the parameter ANOVA (*P*-value < 0.05). NA is reported in the Parameter 3 column when a relationship is modeled with only two parameters. The functional form of the model used to fit the parameters is provided for each relationship

Wildtype mice also exhibit higher coverage compared to KMO-/- mice (Fig. 6B), both ending at a higher final coverage (parameter *a*, *P*-value < 0.0001) and approaching their final coverage level more slowly (parameter *b*, *P*-value = 0.0007), however there is no main age-effect on coverage. While there were only main effects in the time versus activity relationship, parameter *a* of the time versus coverage relationship indicates that there is a statistically significant interaction of genotype and age (*P*-value = 0.0267), where young wildtype mice have higher final coverage levels than either their genotype or age alone would predict.

In the percent coverage versus activity relationship (Fig. 6C), old mice start with higher activity than middle-aged (*P*-value < 0.0001) or young (*P*-value < 0.0001) mice (parameter *a*). In addition to this age-effect, there are both age- and genotype-effects in the final activity level (parameter *c*), with wildtype mice ending at higher activity than KMO-/- mice (*P*-value = 0.0008), and young mice ending at higher activity than middle-aged (*P*-value = 0.0358) or old (*P*-value = 0.0002) mice. Finally, there is both an age and an interactive effect in the rate at which activity decreases as a function of percent coverage. Old mice decrease activity, or habituate, slower than young mice (*P*-value = 0.0140), but this effect is only seen in wildtype mice (interaction *P*-value = 0.0105).

The differences in activity levels are also apparent in the degree of directional persistence that the mice exhibit (Fig. 6D). Wildtype mice start with higher P_++_ than KMO-/- mice (parameter *b*, *P*-value = 0.0032), and decrease their P_++_ more slowly (parameter *a*, *P*-value = 0.0032). Old mice also start with higher P_++_ than middle-aged (*P*-value = 0.0065) or young (*P*-value < 0.0001) mice. Additionally, old mice decrease their P_++_ more quickly than middle-aged (*P*-value < 0.0001), or young (*P*-value < 0.0001) mice, and middle-aged mice decrease their P_++_ more quickly than young mice (*P*-value = 0.0368) as well. In addition to these main effects, there are interactive effects for both initial P_++_ (*P*-value = 0.0007) and rate of P_++_ decrease (*P*-value = 0.0007). For parameter *b*, it is only KMO mice that show higher initial P_++_, and for parameter *c*, old KMO-/- mice show an even faster decrease in P_++_ than their age and genotype alone would predict, indicating faster habituation in KMO-/- old mice.

Interestingly, the decrease in P_++_ that both *Drosophila* and mice exhibit may be due to different behaviors. In *Drosophila*, as P_++_ decreases, it is primarily P_+0_ that increases, meaning that flies increase the frequency of pauses as they habituate to the novel arena, and it is these pauses that provide the opportunity for P_0+_ or P_00_ to occur. On the other hand, in mice, as P_++_ decreases, it is primarily P_+-_ that increases (Fig. 6E), meaning that mice change direction more frequently as they habituate to the novel arena. This also means there are fewer pauses and fewer opportunities for P_0+_ or P_00_ to occur.

The patterns seen in the percent coverage versus P_+-_ relationship parallel those in the percent coverage versus P_++_ relationship. Here, wildtype mice start with lower P_+-_ than KMO-/- mice (*P*-value = 0.0009) and increase their P_+-_ more slowly (*P*-value = 0.0272). Age effects are also seen, with old mice starting at a lower P_+-_ than middle-aged (*P*-value = 0.0244) or young (*P*-value < 0.0001) mice. Old mice also increase their P_+-_ faster than middle-aged (*P*-value < 0.0001) or young (*P*-value < 0.0001) mice. Additionally, middle-aged mice increase their P_+-_ faster than young mice (*P*-value = 0.0243). These age-effects are only seen in wildtype mice due to the interaction between age and genotype (*P*-value = 0.0018 for parameter *a*, and *P*-value = 0.0024 for parameter *b*). The differences between flies and mice in how directional persistence changes over time may reflect different exploratory strategies, and more work should be done to disentangle the causes and consequences of these behavioral differences.

In summary, we found that mice in their old age display higher levels of exploration, with a slower rate of habituation compared to younger and middle-aged mice. These older mice also show defects in initial P_++_ and in P_++_ decay compared to the younger mice, which again reflect a slowing of novelty habituation with aging. Interestingly, these age-related losses in the rate of habituation appear to require Kynurenine 3-monooxygenase activity, as the changes were absent or greatly diminished in the KMO-/- mice. We hypothesize that reduced levels of oxidative and neurotoxic kynurenine pathway (KP) metabolites in KMO-/- mice, such as 3-hydroxykynurenine and quinolinic acid, along with increased levels of the neuroprotective KP metabolite kynurenic acid, contribute to preserving this conserved form of non-associative learning in aging (Parrott et al. 2016; Garrison et al., 2018).

### Additional Use Cases and Future Directions

Here we have demonstrated that *opynfield* can be employed on dense tracking data to accurately calculate and quantify group differences in coverage and directional persistence, leading to a more nuanced understanding of exploratory behavior in Drosophila spp. In addition to its demonstrated use on *Drosophila* species, we have also tested the *opynfield* package on tracking data from open field experiments with mice, indicating that *opynfield* has the potential for widespread adoption beyond the realm of *Drosophila* research.

The package is currently configured to support tracking data from circular arenas of any size, and supports six input file formats from three commonly used tracking software programs. To expand *opynfield*’s use, several opportunities for further development exist. First, the package could be expanded to accommodate data from additional tracking systems, making it more accessible to a wider range of users. Additionally, the package could be enhanced to support additional arena shapes, such as the rectangular arenas commonly employed in rodent studies, by applying a mapping function to the coordinates from a rectangular arena to a circular arena shape.

Beyond data compatibility, *opynfield* could also be expanded to incorporate additional statistical analyses. Currently, the package offers one-way MANOVA and one-way ANOVA for group analyses but could be expanded to include two-way versions of those tests.

While these future additions will enhance the ease of use of the *opynfield* package, it is worth noting how current users can bypass these limitations in the interim. Firstly, users who have data in non-supported file formats could reformat their data into simple CSV files with columns for time, x coordinate, and y coordinate, and read them in as “EthoML” files, as described in the documentation. Additionally, users who have a two-factor experimental design can import the parameter files generated by *opynfield* into a statistical software to carry out the statistical tests, as demonstrated in the mouse analysis above.

## Conclusions

The Python package *opynfield* is a novel software package developed to address the challenges of analyzing open field data by calculating complex measures of exploration using full density tracking data. To our knowledge, available proprietary software solutions offer only basic visualizations of individual subjects’ time versus activity profiles, and do not support statistical analyses of more complex behavioral measures. In the absence of software tools, researchers have relied on single-use analyses that may not be comparable across studies.

In contrast to this, we believe that *opynfield* is positioned to become a critical tool for advancing behavioral biology, helping to standardize analyses, and fostering reproducibility in the field. The *opynfield* package calculates more specific individual-level measures of exploration and supports group-level insights, further data visualization, and robust statistical comparisons.

In addition to these benefits, *opynfield* enables users to make more specific behavioral insights. For example, researchers can use *opynfield* to distinguish if a specific treatment or genotype impacts general activity levels, neophilia, arousal, or learning capabilities. These insights are vital for understanding trait and state-dependent responses to novel stimuli and for studying novelty habituation patterns during exploration.

Through this package, researchers can gain faster, more accurate, and more nuanced insights into open field exploration behavior, paving the way for discoveries in both model organisms and broader study systems.

## Supplementary Information

Below is the link to the electronic supplementary material.

Supplementary file1 List of opynfield’s dependencies (PDF 12 KB)

Supplementary file2 (PDF 16 KB)

Supplementary file3 Full statistical analysis of mouse exploration data (PDF 190 KB)

## Data Availability

Information Sharing Statement opynfield is open-source and freely available. The latest version of opynfield can be accessed on GitHub: https://github.com/EllenMcMullen/opynfield, and the version used in this paper is archived on Zenodo (https://doi.org/10.5281/zenodo.15794680). The datasets used in this paper are included in the software package.
