# Supplementary material for: opynfield: An Open-Source Python Package for the Analysis of Open Field Exploration Data
Source: Neuroinformatics. 2025 Dec 10;23(4):58. doi: 10.1007/s12021-025-09753-2 (PMC12696104; doi:10.1007/s12021-025-09753-2)
Supplement: Supplementary file 1 — Supplementary file1 List of opynfield’s dependencies (PDF 12 KB) [file 12021_2025_9753_MOESM1_ESM.pdf]

opynfield **dependencies:**

- python = ">=3.9,<3.12"
- pandas = "^2.0.1"
- scipy = "^1.10.1"
- openpyxl = "^3.1.2"
- xlrd = "^2.0.1"
- numba = "^0.57.0"
- statsmodels = "^0.14.0"
- matplotlib = "^3.7.1"
- jupyter = "^1.0.0"
