# Supplementary material for: opynfield: An Open-Source Python Package for the Analysis of Open Field Exploration Data
Source: Neuroinformatics. 2025 Dec 10;23(4):58. doi: 10.1007/s12021-025-09753-2 (PMC12696104; doi:10.1007/s12021-025-09753-2)
Supplement: Supplementary file 2 — Supplementary file2 (PDF 16 KB) [file 12021_2025_9753_MOESM2_ESM.pdf]

Example installation and workflow using the package manager conda

1. On the *opynfield* GitHub Page click on Code -> Download ZIP
2. On the *opynfield* GitHub Page, navigate to docs / source / tutorial.ipynb, and click download raw file
3. Open your terminal or command line interface
4. Run: conda create -n opynfield
    a. This will create an environment called opynfield, to house the software and all its dependencies without interfering with other packages or versions you may be using
5. Run: conda activate opynfield
6. Run: conda install python=3.11
7. Run: pip install opynfield-main.zip
8. Run: jupyter-notebook
9. In the notebook file browser, select the tutorial from your downloads, and edit the file to your needs
    a. The notebook will be connected to your opynfield environment, and have access to the correct version of Python, *opynfield*, and all its dependencies
