# Supplementary material for: opynfield: An Open-Source Python Package for the Analysis of Open Field Exploration Data
Source: Neuroinformatics. 2025 Dec 10;23(4):58. doi: 10.1007/s12021-025-09753-2 (PMC12696104; doi:10.1007/s12021-025-09753-2)
Supplement: Supplementary file 3 — Supplementary file3 Full statistical analysis of mouse exploration data (PDF 190 KB) [file 12021_2025_9753_MOESM3_ESM.pdf]

Analysis4TwoWayANOVA

Ellen McMullen

2025-06-17

Load packages:

```
library(stringr)
library(multcomp)
```

```
## Loading required package: mvtnorm

## Loading required package: survival

## Loading required package: TH.data

## Loading required package: MASS

##
## Attaching package: 'TH.data'

## The following object is masked from 'package:MASS':
##
##     geyser
```

Read in data:

```
all_params = read.csv(
  '../ExpectedResultsAnalysis4/Individuals/CombinedGroups/Models/CombinedGroups_IndividualModels.csv')
```

Split the group names (consisting of the genotype and age labels) into two factors

```
all_params[c('genotype', 'age')] = str_split_fixed(all_params$group, ' ', 2)
```

Two-Way MANOVA (Full Test):

```
# manova
two_way_manova =  manova(cbind(time_activity_parameter_0,
                               time_activity_parameter_1,
                               time_activity_parameter_2,
                               time_coverage_parameter_0,
                               time_coverage_parameter_1,
                               time_p_plus_plus_given_plus_parameter_0,
```

```
                                        time_p_plus_plus_given_plus_parameter_1,
                                        time_p_plus_minus_given_plus_parameter_0,
                                        time_p_plus_minus_given_plus_parameter_1,
                                        time_p_plus_zero_given_plus_parameter_0,
                                        time_p_plus_zero_given_plus_parameter_1,
                                        time_p_zero_plus_given_zero_parameter_0,
                                        time_p_zero_plus_given_zero_parameter_1,
                                        time_p_zero_zero_given_zero_parameter_0,
                                        time_p_zero_zero_given_zero_parameter_1,
                                        percent_coverage_activity_parameter_0,
                                        percent_coverage_activity_parameter_1,
                                        percent_coverage_activity_parameter_2,
                                        percent_coverage_p_plus_plus_given_plus_parameter_0,
                                        percent_coverage_p_plus_plus_given_plus_parameter_1,
                                        percent_coverage_p_plus_minus_given_plus_parameter_0,
                                        percent_coverage_p_plus_minus_given_plus_parameter_1,
                                        percent_coverage_p_plus_zero_given_plus_parameter_0,
                                        percent_coverage_p_plus_zero_given_plus_parameter_1,
                                        percent_coverage_p_zero_plus_given_zero_parameter_0,
                                        percent_coverage_p_zero_plus_given_zero_parameter_1,
                                        percent_coverage_p_zero_zero_given_zero_parameter_0,
                                        percent_coverage_p_zero_zero_given_zero_parameter_1)
                                  ~ genotype*age,
                                  data = all_params)
summary(two_way_manova)
```

```
##                 Df  Pillai approx F num Df den Df    Pr(>F)
## genotype         1 0.47484   4.7146     28    146 2.559e-10 ***
## age              2 0.89513   4.2534     56    294 < 2.2e-16 ***
## genotype:age     2 0.40175   1.3197     56    294   0.07611 .
## Residuals      173
## ---
## Signif. codes:  0 '***' 0.001 '**' 0.01 '*' 0.05 '.' 0.1 ' ' 1
```

Now for each relationship of interest, we want to run the MANOVA sub-test:

```
subtest1 =  manova(cbind(time_activity_parameter_0,
                         time_activity_parameter_1,
                         time_activity_parameter_2)
                   ~ genotype*age,
                   data = all_params)
summary(subtest1)
```

```
##                 Df  Pillai approx F num Df den Df    Pr(>F)
## genotype         1 0.19076  14.0649      3    179  2.84e-08 ***
## age              2 0.45745  17.7933      6    360 < 2.2e-16 ***
## genotype:age     2 0.02818   0.8574      6    360    0.5265
## Residuals      181
## ---
## Signif. codes:  0 '***' 0.001 '**' 0.01 '*' 0.05 '.' 0.1 ' ' 1
```

Since the MANOVA sub-test is significant, we want to run follow-up ANOVAs on each parameter

```r
# time vs activity A
mod1 =  aov(time_activity_parameter_0 ~ genotype*age, data = all_params)
summary(mod1)
```

```
##                Df Sum Sq Mean Sq F value   Pr(>F)    
## genotype        1   5.25    5.25   4.117   0.0439 *  
## age             2  71.85   35.92  28.197 2.19e-11 ***
## genotype:age    2   3.41    1.70   1.337   0.2652    
## Residuals     181 230.60    1.27                     
## ---
## Signif. codes:  0 '***' 0.001 '**' 0.01 '*' 0.05 '.' 0.1 ' ' 1
```

```r
# time vs activity B
mod2 =  aov(time_activity_parameter_1 ~ genotype*age, data = all_params)
summary(mod2)
```

```
##                Df Sum Sq Mean Sq F value   Pr(>F)    
## genotype        1    0.7    0.73   0.343    0.559    
## age             2  111.7   55.84  26.368 8.92e-11 ***
## genotype:age    2    4.6    2.31   1.092    0.338    
## Residuals     181  383.3    2.12                     
## ---
## Signif. codes:  0 '***' 0.001 '**' 0.01 '*' 0.05 '.' 0.1 ' ' 1
```

```r
# time vs activity C
mod3 =  aov(time_activity_parameter_2 ~ genotype*age, data = all_params)
summary(mod3)
```

```
##                Df Sum Sq Mean Sq F value   Pr(>F)    
## genotype        1  10.10  10.102  35.987 1.06e-08 ***
## age             2   4.87   2.435   8.675 0.000253 ***
## genotype:age    2   0.29   0.147   0.524 0.593295    
## Residuals     181  50.81   0.281                     
## ---
## Signif. codes:  0 '***' 0.001 '**' 0.01 '*' 0.05 '.' 0.1 ' ' 1
```

We also want to run a Tukey's HSD test as a post-hoc to detect which groups are different from one another, and the direction of the difference.

```r
# time vs activity A post hoc
ph1 = TukeyHSD(aov(time_activity_parameter_0 ~ age+genotype, data = all_params),
               which = "age")
ph1
```

```
##   Tukey multiple comparisons of means
##     95% family-wise confidence level
##
## Fit: aov(formula = time_activity_parameter_0 ~ age + genotype, data = all_params)
##
## $age
##                          diff        lwr        upr     p adj
```

```
## Old-Middle Aged    1.3644839  0.8618858  1.8670821 0.0000000
## Young-Middle Aged -0.1866424 -0.6362220  0.2629373 0.5898131
## Young-Old         -1.5511263 -2.0676907 -1.0345618 0.0000000
```

```
ph2 = TukeyHSD(aov(time_activity_parameter_0 ~ age+genotype, data = all_params),
               which = "genotype")
ph2
```

```
##   Tukey multiple comparisons of means
##     95% family-wise confidence level
##
## Fit: aov(formula = time_activity_parameter_0 ~ age + genotype, data = all_params)
##
## $genotype
##             diff         lwr       upr     p adj
## WT-KMO 0.2883761 -0.03793618 0.6146884 0.0829021
```

```
# time vs activity B post hoc
ph3 = TukeyHSD(aov(time_activity_parameter_1 ~ age+genotype, data = all_params),
               which = "age")
ph3
```

```
##   Tukey multiple comparisons of means
##     95% family-wise confidence level
##
## Fit: aov(formula = time_activity_parameter_1 ~ age + genotype, data = all_params)
##
## $age
##                         diff        lwr        upr     p adj
## Old-Middle Aged    0.2314979 -0.4156354  0.8786312 0.6753681
## Young-Middle Aged -1.5202300 -2.0990980 -0.9413620 0.0000000
## Young-Old         -1.7517279 -2.4168439 -1.0866119 0.0000000
```

```
ph4 = TukeyHSD(aov(time_activity_parameter_1 ~ age+genotype, data = all_params),
               which = "genotype")
ph4
```

```
##   Tukey multiple comparisons of means
##     95% family-wise confidence level
##
## Fit: aov(formula = time_activity_parameter_1 ~ age + genotype, data = all_params)
##
## $genotype
##             diff        lwr      upr     p adj
## WT-KMO 0.1224551 -0.2976968 0.542607 0.5659685
```

```
# time vs activity C post hoc
ph5 = TukeyHSD(aov(time_activity_parameter_2 ~ age+genotype, data = all_params),
               which = "age")
ph5
```

```
##   Tukey multiple comparisons of means
##     95% family-wise confidence level
##
## Fit: aov(formula = time_activity_parameter_2 ~ age + genotype, data = all_params)
##
## $age
##                           diff         lwr        upr     p adj
## Old-Middle Aged   -0.1613781 -0.39624541 0.07348924 0.2382987
## Young-Middle Aged  0.2424592  0.03236773 0.45255063 0.0191281
## Young-Old          0.4038373  0.16244340 0.64523114 0.0003231
```

```
ph6 = TukeyHSD(aov(time_activity_parameter_2 ~ age+genotype, data = all_params),
               which = "genotype")
ph6
```

```
##   Tukey multiple comparisons of means
##     95% family-wise confidence level
##
## Fit: aov(formula = time_activity_parameter_2 ~ age + genotype, data = all_params)
##
## $genotype
##             diff       lwr      upr p adj
## WT-KMO 0.4695282 0.3170403 0.622016     0
```

Repeat for other relationships:

```
subtest2 =  manova(cbind(time_coverage_parameter_0,
                         time_coverage_parameter_1)
                   ~ genotype*age,
                   data = all_params)
summary(subtest2)
```

```
##                Df   Pillai approx F num Df den Df    Pr(>F)
## genotype        1 0.129669  13.4090      2    180 3.729e-06 ***
## age             2 0.043071   1.9919      4    362   0.09520 .
## genotype:age    2 0.047698   2.2110      4    362   0.06733 .
## Residuals     181
## ---
## Signif. codes:  0 '***' 0.001 '**' 0.01 '*' 0.05 '.' 0.1 ' ' 1
```

```
# time vs coverage A
mod4 =  aov(time_coverage_parameter_0 ~ genotype*age, data = all_params)
summary(mod4)
```

```
##                Df Sum Sq Mean Sq F value  Pr(>F)
## genotype        1  311.7  311.73  26.644 6.4e-07 ***
## age             2   19.3    9.65   0.825  0.4400
## genotype:age    2   86.5   43.25   3.696  0.0267 *
## Residuals     181 2117.7   11.70
## ---
## Signif. codes:  0 '***' 0.001 '**' 0.01 '*' 0.05 '.' 0.1 ' ' 1
```

```r
# time vs coverage B
mod5 =  aov(time_coverage_parameter_1 ~ genotype*age, data = all_params)
summary(mod5)
```

```
##                Df    Sum Sq   Mean Sq F value   Pr(>F)
## genotype        1 0.0002025 2.025e-04  11.934 0.000687 ***
## age             2 0.0000848 4.240e-05   2.499 0.085010 .
## genotype:age    2 0.0000389 1.947e-05   1.148 0.319726
## Residuals     181 0.0030713 1.697e-05
## ---
## Signif. codes:  0 '***' 0.001 '**' 0.01 '*' 0.05 '.' 0.1 ' ' 1
```

```r
# time vs coverage A post hoc
ph7 = TukeyHSD(aov(time_coverage_parameter_0 ~ age+genotype, data = all_params),
               which = "age")
ph7
```

```
##   Tukey multiple comparisons of means
##     95% family-wise confidence level
##
## Fit: aov(formula = time_coverage_parameter_0 ~ age + genotype, data = all_params)
##
## $age
##                          diff       lwr       upr     p adj
## Old-Middle Aged    -0.4371339 -1.979653 1.1053847 0.7814117
## Young-Middle Aged -0.8046015 -2.184402 0.5751987 0.3546003
## Young-Old          -0.3674675 -1.952850 1.2179149 0.8477862
```

```r
ph8 = TukeyHSD(aov(time_coverage_parameter_0 ~ age+genotype, data = all_params),
               which = "genotype")
ph8
```

```
##   Tukey multiple comparisons of means
##     95% family-wise confidence level
##
## Fit: aov(formula = time_coverage_parameter_0 ~ age + genotype, data = all_params)
##
## $genotype
##             diff       lwr      upr   p adj
## WT-KMO -2.564101 -3.565583 -1.56262 1.1e-06
```

```r
# time vs coverage B post hoc
ph9 = TukeyHSD(aov(time_coverage_parameter_1 ~ age+genotype, data = all_params),
               which = "age")
ph9
```

```
##   Tukey multiple comparisons of means
##     95% family-wise confidence level
##
## Fit: aov(formula = time_coverage_parameter_1 ~ age + genotype, data = all_params)
##
```

```
## $age
##                               diff           lwr          upr     p adj
## Old-Middle Aged   -0.0011344761 -0.0029668185 0.0006978662 0.3111838
## Young-Middle Aged  0.0005538695 -0.0010851812 0.0021929202 0.7044257
## Young-Old          0.0016883456 -0.0001949143 0.0035716054 0.0889185
```

```
ph10 = TukeyHSD(aov(time_coverage_parameter_1 ~ age+genotype, data = all_params),
                which = "genotype")
ph10
```

```
##   Tukey multiple comparisons of means
##     95% family-wise confidence level
##
## Fit: aov(formula = time_coverage_parameter_1 ~ age + genotype, data = all_params)
##
## $genotype
##                diff          lwr         upr     p adj
## WT-KMO 0.002118491 0.0009288411 0.003308141 0.0005573
```

```
subtest3 =  manova(cbind(time_p_plus_plus_given_plus_parameter_0,
                         time_p_plus_plus_given_plus_parameter_1)
                   ~ genotype*age,
                   data = all_params)
summary(subtest3)
```

```
##                Df  Pillai approx F num Df den Df    Pr(>F)
## genotype        1 0.31687   41.747      2    180 1.274e-15 ***
## age             2 0.22742   11.611      4    362 7.005e-09 ***
## genotype:age    2 0.05179    2.406      4    362   0.04926 *
## Residuals     181
## ---
## Signif. codes:  0 '***' 0.001 '**' 0.01 '*' 0.05 '.' 0.1 ' ' 1
```

```
# time vs p++ A
mod6 =  aov(time_p_plus_plus_given_plus_parameter_0 ~ genotype*age,
            data = all_params)
summary(mod6)
```

```
##                Df    Sum Sq   Mean Sq F value   Pr(>F)
## genotype        1 1.320e-07 1.324e-07   3.430   0.0656 .
## age             2 1.668e-06 8.341e-07  21.615 3.82e-09 ***
## genotype:age    2 3.070e-07 1.537e-07   3.984   0.0203 *
## Residuals     181 6.984e-06 3.860e-08
## ---
## Signif. codes:  0 '***' 0.001 '**' 0.01 '*' 0.05 '.' 0.1 ' ' 1
```

```
# time vs p++ B
mod7 =  aov(time_p_plus_plus_given_plus_parameter_1 ~ genotype*age,
            data = all_params)
summary(mod7)
```

```
##                Df Sum Sq Mean Sq F value   Pr(>F)
## genotype       1 0.2937 0.29374  42.775 6.08e-10 ***
## age            2 0.0426 0.02132   3.105   0.0472 *
## genotype:age   2 0.0434 0.02169   3.158   0.0449 *
## Residuals    181 1.2430 0.00687
## ---
## Signif. codes:  0 '***' 0.001 '**' 0.01 '*' 0.05 '.' 0.1 ' ' 1
```

```r
# time vs p++ A post hoc
ph11 = TukeyHSD(aov(time_p_plus_plus_given_plus_parameter_0 ~ age+genotype,
                    data = all_params), which = "age")
ph11
```

```
##   Tukey multiple comparisons of means
##     95% family-wise confidence level
##
## Fit: aov(formula = time_p_plus_plus_given_plus_parameter_0 ~ age + genotype, data = all_params)
##
## $age
##                              diff           lwr           upr     p adj
## Old-Middle Aged   -1.558733e-04 -2.445939e-04 -6.715268e-05 0.0001491
## Young-Middle Aged  9.154771e-05  1.218614e-05  1.709093e-04 0.0191932
## Young-Old          2.474210e-04  1.562350e-04  3.386070e-04 0.0000000
```

```r
ph12 = TukeyHSD(aov(time_p_plus_plus_given_plus_parameter_0 ~ age+genotype,
                    data = all_params), which = "genotype")
ph12
```

```
##   Tukey multiple comparisons of means
##     95% family-wise confidence level
##
## Fit: aov(formula = time_p_plus_plus_given_plus_parameter_0 ~ age + genotype, data = all_params)
##
## $genotype
##                diff          lwr          upr     p adj
## WT-KMO 5.826844e-05 6.665053e-07 0.0001158704 0.0474343
```

```r
# time vs p++ B post hoc
ph13 = TukeyHSD(aov(time_p_plus_plus_given_plus_parameter_1 ~ age+genotype,
                    data = all_params), which = "age")
ph13
```

```
##   Tukey multiple comparisons of means
##     95% family-wise confidence level
##
## Fit: aov(formula = time_p_plus_plus_given_plus_parameter_1 ~ age + genotype, data = all_params)
##
## $age
##                          diff         lwr         upr     p adj
## Old-Middle Aged    0.00433393 -0.03292949 0.041597354 0.9592302
## Young-Middle Aged -0.02965657 -0.06298912 0.003675979 0.0921526
## Young-Old         -0.03399050 -0.07228941 0.004308408 0.0932275
```

```
ph14 = TukeyHSD(aov(time_p_plus_plus_given_plus_parameter_1 ~ age+genotype,
                    data = all_params), which = "genotype")
ph14
```

```
##   Tukey multiple comparisons of means
##     95% family-wise confidence level
##
## Fit: aov(formula = time_p_plus_plus_given_plus_parameter_1 ~ age + genotype, data = all_params)
##
## $genotype
##              diff        lwr       upr p adj
## WT-KMO 0.07922986 0.05503655 0.1034232     0
```

```
subtest4 =  manova(cbind(time_p_plus_minus_given_plus_parameter_0,
                         time_p_plus_minus_given_plus_parameter_1)
                   ~ genotype*age,
                   data = all_params)
summary(subtest4)
```

```
##                 Df   Pillai approx F num Df den Df    Pr(>F)
## genotype         1 0.300079   38.586      2    180 1.133e-14 ***
## age              2 0.210639   10.653      4    362 3.583e-08 ***
## genotype:age     2 0.048793    2.263      4    362   0.06196 .
## Residuals      181
## ---
## Signif. codes:  0 '***' 0.001 '**' 0.01 '*' 0.05 '.' 0.1 ' ' 1
```

```
# time vs p+- A
mod8 =  aov(time_p_plus_minus_given_plus_parameter_0 ~ genotype*age,
            data = all_params)
summary(mod8)
```

```
##                 Df    Sum Sq   Mean Sq F value   Pr(>F)
## genotype         1 2.700e-08 2.680e-08   0.983   0.3229
## age              2 1.010e-06 5.052e-07  18.493 4.92e-08 ***
## genotype:age     2 2.170e-07 1.083e-07   3.963   0.0207 *
## Residuals      181 4.945e-06 2.730e-08
## ---
## Signif. codes:  0 '***' 0.001 '**' 0.01 '*' 0.05 '.' 0.1 ' ' 1
```

```
# time vs p+- B
mod9 =  aov(time_p_plus_minus_given_plus_parameter_1 ~ genotype*age,
            data = all_params)
summary(mod9)
```

```
##                 Df Sum Sq Mean Sq F value   Pr(>F)
## genotype         1 0.2230 0.22299  41.261 1.14e-09 ***
## age              2 0.0359 0.01793   3.317   0.0385 *
## genotype:age     2 0.0325 0.01624   3.005   0.0520 .
## Residuals      181 0.9782 0.00540
## ---
## Signif. codes:  0 '***' 0.001 '**' 0.01 '*' 0.05 '.' 0.1 ' ' 1
```

```r
# time vs p+- A post hoc
ph15 = TukeyHSD(aov(time_p_plus_minus_given_plus_parameter_0 ~ age+genotype,
                    data = all_params), which = "age")
ph15
```

```
##   Tukey multiple comparisons of means
##     95% family-wise confidence level
##
## Fit: aov(formula = time_p_plus_minus_given_plus_parameter_0 ~ age + genotype, data = all_params)
##
## $age
##                              diff           lwr           upr     p adj
## Old-Middle Aged     1.135563e-04  0.0000389152  1.881975e-04 0.0012087
## Young-Middle Aged -7.990859e-05 -0.0001466759 -1.314126e-05 0.0143407
## Young-Old         -1.934649e-04 -0.0002701802 -1.167497e-04 0.0000000
```

```r
ph16 = TukeyHSD(aov(time_p_plus_minus_given_plus_parameter_0 ~ age+genotype,
                    data = all_params), which = "genotype")
ph16
```

```
##   Tukey multiple comparisons of means
##     95% family-wise confidence level
##
## Fit: aov(formula = time_p_plus_minus_given_plus_parameter_0 ~ age + genotype, data = all_params)
##
## $genotype
##                diff           lwr          upr     p adj
## WT-KMO -2.759671e-05 -7.605754e-05 2.086412e-05 0.2626717
```

```r
# time vs p+- B post hoc
ph17 = TukeyHSD(aov(time_p_plus_minus_given_plus_parameter_1 ~ age+genotype,
                    data = all_params), which = "age")
ph17
```

```
##   Tukey multiple comparisons of means
##     95% family-wise confidence level
##
## Fit: aov(formula = time_p_plus_minus_given_plus_parameter_1 ~ age + genotype, data = all_params)
##
## $age
##                           diff          lwr        upr     p adj
## Old-Middle Aged   -0.001431415 -0.034462072 0.03159924 0.9942359
## Young-Middle Aged  0.028075762 -0.001470530 0.05762205 0.0664202
## Young-Old          0.029507177 -0.004441343 0.06345570 0.1025257
```

```r
ph18 = TukeyHSD(aov(time_p_plus_minus_given_plus_parameter_1 ~ age+genotype,
                    data = all_params), which = "genotype")
ph18
```

```
##   Tukey multiple comparisons of means
##     95% family-wise confidence level
```

```
##
## Fit: aov(formula = time_p_plus_minus_given_plus_parameter_1 ~ age + genotype, data = all_params)
##
## $genotype
##                 diff        lwr         upr p adj
## WT-KMO -0.06912192 -0.0905671 -0.04767673     0
```

```
subtest5 =  manova(cbind(time_p_plus_zero_given_plus_parameter_0,
                         time_p_plus_zero_given_plus_parameter_1)
                   ~ genotype*age,
                   data = all_params)
summary(subtest5)
```

```
##                Df   Pillai approx F num Df den Df    Pr(>F)
## genotype        1 0.294920   37.645      2    180 2.195e-14 ***
## age             2 0.210979   10.673      4    362 3.468e-08 ***
## genotype:age    2 0.035339    1.628      4    362    0.1666
## Residuals     181
## ---
## Signif. codes:  0 '***' 0.001 '**' 0.01 '*' 0.05 '.' 0.1 ' ' 1
```

```
# time vs p+0 A
mod10 =  aov(time_p_plus_zero_given_plus_parameter_0 ~ genotype*age,
             data = all_params)
summary(mod10)
```

```
##                Df    Sum Sq   Mean Sq F value   Pr(>F)
## genotype        1 5.720e-08 5.720e-08  37.061 6.69e-09 ***
## age             2 7.372e-08 3.686e-08  23.882 6.24e-10 ***
## genotype:age    2 4.580e-09 2.290e-09   1.482     0.23
## Residuals     181 2.794e-07 1.540e-09
## ---
## Signif. codes:  0 '***' 0.001 '**' 0.01 '*' 0.05 '.' 0.1 ' ' 1
```

```
# time vs p+0 B
mod11 =  aov(time_p_plus_zero_given_plus_parameter_1 ~ genotype*age,
             data = all_params)
summary(mod11)
```

```
##                Df   Sum Sq  Mean Sq F value   Pr(>F)
## genotype        1 0.003451 0.003451  34.603 1.92e-08 ***
## age             2 0.000126 0.000063   0.632    0.533
## genotype:age    2 0.000376 0.000188   1.886    0.155
## Residuals     181 0.018051 0.000100
## ---
## Signif. codes:  0 '***' 0.001 '**' 0.01 '*' 0.05 '.' 0.1 ' ' 1
```

```
# time vs p+0 A post hoc
ph19 = TukeyHSD(aov(time_p_plus_zero_given_plus_parameter_0 ~ age+genotype,
                    data = all_params), which = "age")
ph19
```

```
##   Tukey multiple comparisons of means
##     95% family-wise confidence level
##
## Fit: aov(formula = time_p_plus_zero_given_plus_parameter_0 ~ age + genotype, data = all_params)
##
## $age
##                            diff           lwr           upr     p adj
## Old-Middle Aged    4.214449e-05  2.463705e-05  5.965193e-05 0.0000002
## Young-Middle Aged -5.181063e-06 -2.084166e-05  1.047954e-05 0.7147116
## Young-Old         -4.732555e-05 -6.531949e-05 -2.933161e-05 0.0000000
```

```
ph20 = TukeyHSD(aov(time_p_plus_zero_given_plus_parameter_0 ~ age+genotype,
                    data = all_params), which = "genotype")
ph20
```

```
##   Tukey multiple comparisons of means
##     95% family-wise confidence level
##
## Fit: aov(formula = time_p_plus_zero_given_plus_parameter_0 ~ age + genotype, data = all_params)
##
## $genotype
##                 diff           lwr           upr p adj
## WT-KMO -3.64212e-05 -4.778792e-05 -2.505447e-05     0
```

```
# time vs p+0 B post hoc
ph21 = TukeyHSD(aov(time_p_plus_zero_given_plus_parameter_1 ~ age+genotype,
                    data = all_params), which = "age")
ph21
```

```
##   Tukey multiple comparisons of means
##     95% family-wise confidence level
##
## Fit: aov(formula = time_p_plus_zero_given_plus_parameter_1 ~ age + genotype, data = all_params)
##
## $age
##                            diff          lwr         upr     p adj
## Old-Middle Aged   -0.0025355717 -0.006995570 0.001924427 0.3731011
## Young-Middle Aged -0.0004930813 -0.004482600 0.003496438 0.9540849
## Young-Old          0.0020424904 -0.002541444 0.006626425 0.5445875
```

```
ph22 = TukeyHSD(aov(time_p_plus_zero_given_plus_parameter_1 ~ age+genotype,
                    data = all_params), which = "genotype")
ph22
```

```
##   Tukey multiple comparisons of means
##     95% family-wise confidence level
##
## Fit: aov(formula = time_p_plus_zero_given_plus_parameter_1 ~ age + genotype, data = all_params)
##
## $genotype
##                diff         lwr          upr p adj
## WT-KMO -0.00850202 -0.01139768 -0.005606362     0
```

```r
subtest6 =  manova(cbind(time_p_zero_plus_given_zero_parameter_0,
                         time_p_zero_plus_given_zero_parameter_1)
                   ~ genotype*age,
                   data = all_params)
summary(subtest6)
```

```
##                 Df   Pillai approx F num Df den Df    Pr(>F)
## genotype         1 0.113158  10.9733      2    172 3.272e-05 ***
## age              2 0.002420   0.1048      4    346    0.9808
## genotype:age     2 0.011857   0.5159      4    346    0.7241
## Residuals      173
## ---
## Signif. codes:  0 '***' 0.001 '**' 0.01 '*' 0.05 '.' 0.1 ' ' 1
```

```r
# time vs p0+ A
mod12 =  aov(time_p_zero_plus_given_zero_parameter_0 ~ genotype*age,
             data = all_params)
summary(mod12)
```

```
##                 Df    Sum Sq   Mean Sq F value  Pr(>F)
## genotype         1 1.021e-05 1.021e-05   7.218 0.00792 **
## age              2 3.300e-07 1.640e-07   0.116 0.89042
## genotype:age     2 2.010e-06 1.005e-06   0.710 0.49286
## Residuals      173 2.446e-04 1.414e-06
## ---
## Signif. codes:  0 '***' 0.001 '**' 0.01 '*' 0.05 '.' 0.1 ' ' 1
## 8 observations deleted due to missingness
```

```r
# time vs p0+ B
mod13 =  aov(time_p_zero_plus_given_zero_parameter_1 ~ genotype*age,
             data = all_params)
summary(mod13)
```

```
##                 Df Sum Sq Mean Sq F value   Pr(>F)
## genotype         1   5.78   5.778  14.963 0.000155 ***
## age              2   0.10   0.051   0.132 0.876225
## genotype:age     2   0.60   0.298   0.773 0.463353
## Residuals      173  66.81   0.386
## ---
## Signif. codes:  0 '***' 0.001 '**' 0.01 '*' 0.05 '.' 0.1 ' ' 1
## 8 observations deleted due to missingness
```

```r
# time vs p0+ A post hoc
ph23 = TukeyHSD(aov(time_p_zero_plus_given_zero_parameter_0 ~ age+genotype,
                    data = all_params), which = "age")
ph23
```

```
##   Tukey multiple comparisons of means
##     95% family-wise confidence level
##
## Fit: aov(formula = time_p_zero_plus_given_zero_parameter_0 ~ age + genotype, data = all_params)
```

```
##
## $age
##                              diff           lwr          upr     p adj
## Old-Middle Aged   -9.773502e-05 -0.0006319765 0.0004365065 0.9021267
## Young-Middle Aged -8.735193e-05 -0.0005726683 0.0003979644 0.9051032
## Young-Old          1.038309e-05 -0.0005446798 0.0005654460 0.9989226
```

```r
ph24 = TukeyHSD(aov(time_p_zero_plus_given_zero_parameter_0 ~ age+genotype,
                    data = all_params), which = "genotype")
ph24
```

```
##   Tukey multiple comparisons of means
##     95% family-wise confidence level
##
## Fit: aov(formula = time_p_zero_plus_given_zero_parameter_0 ~ age + genotype, data = all_params)
##
## $genotype
##                 diff           lwr           upr     p adj
## WT-KMO -0.0004764384 -0.0008269648 -0.0001259119 0.0080066
```

```r
# time vs p0+ B post hoc
ph25 = TukeyHSD(aov(time_p_zero_plus_given_zero_parameter_1 ~ age+genotype,
                    data = all_params), which = "age")
ph25
```

```
##   Tukey multiple comparisons of means
##     95% family-wise confidence level
##
## Fit: aov(formula = time_p_zero_plus_given_zero_parameter_1 ~ age + genotype, data = all_params)
##
## $age
##                          diff        lwr       upr     p adj
## Old-Middle Aged   0.044771109 -0.2345046 0.3240469 0.9239365
## Young-Middle Aged 0.050210677 -0.2034894 0.3039107 0.8864513
## Young-Old         0.005439568 -0.2847206 0.2955997 0.9989179
```

```r
ph26 = TukeyHSD(aov(time_p_zero_plus_given_zero_parameter_1 ~ age+genotype,
                    data = all_params), which = "genotype")
ph26
```

```
##   Tukey multiple comparisons of means
##     95% family-wise confidence level
##
## Fit: aov(formula = time_p_zero_plus_given_zero_parameter_1 ~ age + genotype, data = all_params)
##
## $genotype
##             diff       lwr       upr    p adj
## WT-KMO 0.3592079 0.1759695 0.5424462 0.000154
```

```r
subtest7 =  manova(cbind(time_p_zero_zero_given_zero_parameter_0,
                         time_p_zero_zero_given_zero_parameter_1)
```

```r
                    ~ genotype*age,
                    data = all_params)
summary(subtest7)
```

```
##                Df   Pillai approx F num Df den Df    Pr(>F)    
## genotype        1 0.101542   9.7196      2    172 0.0001002 ***
## age             2 0.031663   1.3914      4    346 0.2364615    
## genotype:age    2 0.012562   0.5468      4    346 0.7015124    
## Residuals     173                                              
## ---
## Signif. codes:  0 '***' 0.001 '**' 0.01 '*' 0.05 '.' 0.1 ' ' 1
```

```r
# time vs p00 A
mod14 =  aov(time_p_zero_zero_given_zero_parameter_0 ~ genotype*age,
             data = all_params)
summary(mod14)
```

```
##                Df    Sum Sq   Mean Sq F value Pr(>F)  
## genotype        1 0.000e+00 6.000e-10   0.003 0.9539  
## age             2 9.200e-07 4.608e-07   2.517 0.0836 .
## genotype:age    2 1.700e-07 8.280e-08   0.452 0.6370  
## Residuals     173 3.167e-05 1.831e-07                 
## ---
## Signif. codes:  0 '***' 0.001 '**' 0.01 '*' 0.05 '.' 0.1 ' ' 1
## 8 observations deleted due to missingness
```

```r
# time vs p00 B
mod15 =  aov(time_p_zero_zero_given_zero_parameter_1 ~ genotype*age,
             data = all_params)
summary(mod15)
```

```
##                Df Sum Sq Mean Sq F value   Pr(>F)    
## genotype        1  0.733  0.7325  13.159 0.000376 ***
## age             2  0.162  0.0808   1.452 0.236870    
## genotype:age    2  0.109  0.0544   0.978 0.378258    
## Residuals     173  9.631  0.0557                     
## ---
## Signif. codes:  0 '***' 0.001 '**' 0.01 '*' 0.05 '.' 0.1 ' ' 1
## 8 observations deleted due to missingness
```

```r
# time vs p00 A post hoc
ph27 = TukeyHSD(aov(time_p_zero_zero_given_zero_parameter_0 ~ age+genotype,
                    data = all_params), which = "age")
ph27
```

```
##   Tukey multiple comparisons of means
##     95% family-wise confidence level
## 
## Fit: aov(formula = time_p_zero_zero_given_zero_parameter_0 ~ age + genotype, data = all_params)
## 
## $age
```

```
##                               diff           lwr          upr     p adj
## Old-Middle Aged    1.793881e-04 -1.254188e-05 3.713181e-04 0.0723800
## Young-Middle Aged  3.971135e-05 -1.346420e-04 2.140646e-04 0.8525115
## Young-Old         -1.396768e-04 -3.390870e-04 5.973347e-05 0.2253076
```

```r
ph28 = TukeyHSD(aov(time_p_zero_zero_given_zero_parameter_0 ~ age+genotype,
                    data = all_params), which = "genotype")
ph28
```

```
##   Tukey multiple comparisons of means
##     95% family-wise confidence level
##
## Fit: aov(formula = time_p_zero_zero_given_zero_parameter_0 ~ age + genotype, data = all_params)
##
## $genotype
##                 diff           lwr          upr     p adj
## WT-KMO -1.001466e-05 -0.0001359437 0.0001159144 0.8754622
```

```r
# time vs p00 B post hoc
ph29 = TukeyHSD(aov(time_p_zero_zero_given_zero_parameter_1 ~ age+genotype,
                    data = all_params), which = "age")
ph29
```

```
##   Tukey multiple comparisons of means
##     95% family-wise confidence level
##
## Fit: aov(formula = time_p_zero_zero_given_zero_parameter_1 ~ age + genotype, data = all_params)
##
## $age
##                          diff         lwr        upr     p adj
## Old-Middle Aged   -0.08218885 -0.18834908 0.02397139 0.1628177
## Young-Middle Aged -0.03494989 -0.13138811 0.06148832 0.6682764
## Young-Old          0.04723895 -0.06305875 0.15753665 0.5700355
```

```r
ph30 = TukeyHSD(aov(time_p_zero_zero_given_zero_parameter_1 ~ age+genotype,
                    data = all_params), which = "genotype")
ph30
```

```
##   Tukey multiple comparisons of means
##     95% family-wise confidence level
##
## Fit: aov(formula = time_p_zero_zero_given_zero_parameter_1 ~ age + genotype, data = all_params)
##
## $genotype
##              diff        lwr         upr     p adj
## WT-KMO -0.1256416 -0.1952955 -0.05598781 0.0004776
```

```r
subtest8 =  manova(cbind(percent_coverage_activity_parameter_0,
                         percent_coverage_activity_parameter_1,
                         percent_coverage_activity_parameter_2)
                   ~ genotype*age,
                   data = all_params)
summary(subtest8)
```

```
##                Df  Pillai approx F num Df den Df    Pr(>F)
## genotype        1 0.14862   10.415      3    179 2.374e-06 ***
## age             2 0.32045   11.448      6    360 1.001e-11 ***
## genotype:age    2 0.06470    2.006      6    360   0.06413 .
## Residuals     181
## ---
## Signif. codes:  0 '***' 0.001 '**' 0.01 '*' 0.05 '.' 0.1 ' ' 1
```

```r
# percent_coverage vs activity A
mod16 =  aov(percent_coverage_activity_parameter_0 ~ genotype*age,
             data = all_params)
summary(mod16)
```

```
##                Df Sum Sq Mean Sq F value   Pr(>F)
## genotype        1   5.68    5.68   3.423   0.0659 .
## age             2 127.05   63.53  38.289 1.36e-14 ***
## genotype:age    2   2.99    1.50   0.902   0.4077
## Residuals     181 300.30    1.66
## ---
## Signif. codes:  0 '***' 0.001 '**' 0.01 '*' 0.05 '.' 0.1 ' ' 1
```

```r
# percent_coverage vs activity B
mod17 =  aov(percent_coverage_activity_parameter_1 ~ genotype*age,
             data = all_params)
summary(mod17)
```

```
##                Df Sum Sq Mean Sq F value Pr(>F)
## genotype        1    0.4   0.380   0.058 0.8107
## age             2   57.9  28.926   4.374 0.0140 *
## genotype:age    2   61.8  30.890   4.671 0.0105 *
## Residuals     181 1197.0   6.613
## ---
## Signif. codes:  0 '***' 0.001 '**' 0.01 '*' 0.05 '.' 0.1 ' ' 1
```

```r
# percent_coverage vs activity C
mod18 =  aov(percent_coverage_activity_parameter_2 ~ genotype*age,
             data = all_params)
summary(mod18)
```

```
##                Df Sum Sq Mean Sq F value   Pr(>F)
## genotype        1   3.12  3.1187  11.591 0.000816 ***
## age             2   4.68  2.3415   8.703 0.000246 ***
## genotype:age    2   0.82  0.4116   1.530 0.219340
## Residuals     181  48.70  0.2691
## ---
## Signif. codes:  0 '***' 0.001 '**' 0.01 '*' 0.05 '.' 0.1 ' ' 1
```

```r
# percent_coverage vs activity A post hoc
ph31 = TukeyHSD(aov(percent_coverage_activity_parameter_0 ~ age+genotype,
                    data = all_params), which = "age")
ph31
```

```
##   Tukey multiple comparisons of means
##     95% family-wise confidence level
##
## Fit: aov(formula = percent_coverage_activity_parameter_0 ~ age + genotype, data = all_params)
##
## $age
##                          diff        lwr        upr     p adj
## Old-Middle Aged     1.7109616  1.1387745  2.2831487 0.0000000
## Young-Middle Aged -0.4018736 -0.9137013  0.1099542 0.1548091
## Young-Old         -2.1128352 -2.7009224 -1.5247480 0.0000000
```

```
ph32 = TukeyHSD(aov(percent_coverage_activity_parameter_0 ~ age+genotype,
                    data = all_params), which = "genotype")
ph32
```

```
##   Tukey multiple comparisons of means
##     95% family-wise confidence level
##
## Fit: aov(formula = percent_coverage_activity_parameter_0 ~ age + genotype, data = all_params)
##
## $genotype
##              diff         lwr       upr     p adj
## WT-KMO 0.2907518 -0.08074118 0.6622448 0.1242692
```

```
# percent_coverage vs activity B post hoc
ph33 = TukeyHSD(aov(percent_coverage_activity_parameter_1 ~ age+genotype,
                    data = all_params), which = "age")
ph33
```

```
##   Tukey multiple comparisons of means
##     95% family-wise confidence level
##
## Fit: aov(formula = percent_coverage_activity_parameter_1 ~ age + genotype, data = all_params)
##
## $age
##                          diff        lwr        upr     p adj
## Old-Middle Aged     0.6111918 -0.5544783  1.7768620 0.4318269
## Young-Middle Aged -0.8265539 -1.8692590  0.2161511 0.1494337
## Young-Old         -1.4377458 -2.6358079 -0.2396837 0.0140260
```

```
ph34 = TukeyHSD(aov(percent_coverage_activity_parameter_1 ~ age+genotype,
                    data = all_params), which = "genotype")
ph34
```

```
##   Tukey multiple comparisons of means
##     95% family-wise confidence level
##
## Fit: aov(formula = percent_coverage_activity_parameter_1 ~ age + genotype, data = all_params)
##
## $genotype
##               diff        lwr       upr     p adj
## WT-KMO 0.07214051 -0.6846719 0.8289529 0.8510298
```

```r
# percent_coverage vs activity C post hoc
ph35 = TukeyHSD(aov(percent_coverage_activity_parameter_2 ~ age+genotype,
                    data = all_params), which = "age")
ph35
```

```
##   Tukey multiple comparisons of means
##     95% family-wise confidence level
##
## Fit: aov(formula = percent_coverage_activity_parameter_2 ~ age + genotype, data = all_params)
##
## $age
##                           diff         lwr        upr     p adj
## Old-Middle Aged   -0.1874725 -0.41868430 0.04373931 0.1370685
## Young-Middle Aged  0.2183558  0.01153428 0.42517738 0.0358140
## Young-Old          0.4058283  0.16819156 0.64346509 0.0002353
```

```r
ph36 = TukeyHSD(aov(percent_coverage_activity_parameter_2 ~ age+genotype,
                    data = all_params), which = "genotype")
ph36
```

```
##   Tukey multiple comparisons of means
##     95% family-wise confidence level
##
## Fit: aov(formula = percent_coverage_activity_parameter_2 ~ age + genotype, data = all_params)
##
## $genotype
##             diff       lwr       upr     p adj
## WT-KMO 0.2639589 0.1138444 0.4140734 0.0006506
```

```r
subtest9 =  manova(cbind(percent_coverage_p_plus_plus_given_plus_parameter_0,
                         percent_coverage_p_plus_plus_given_plus_parameter_1)
                   ~ genotype*age,
                   data = all_params)
summary(subtest9)
```

```
##                 Df   Pillai approx F num Df den Df    Pr(>F)
## genotype         1 0.289637   36.696      2    180 4.298e-14 ***
## age              2 0.256165   13.294      4    362 4.069e-10 ***
## genotype:age     2 0.087299    4.131      4    362  0.002761 **
## Residuals      181
## ---
## Signif. codes:  0 '***' 0.001 '**' 0.01 '*' 0.05 '.' 0.1 ' ' 1
```

```r
# percent_coverage vs p++ A
mod19 =  aov(percent_coverage_p_plus_plus_given_plus_parameter_0 ~ genotype*age,
             data = all_params)
summary(mod19)
```

```
##                 Df Sum Sq Mean Sq F value   Pr(>F)
## genotype         1 0.1448  0.1448   8.941 0.003177 **
## age              2 0.9477  0.4739  29.267 9.71e-12 ***
```

```
## genotype:age   2 0.2449  0.1225   7.563 0.000701 ***
## Residuals    181 2.9306  0.0162
## ---
## Signif. codes:  0 '***' 0.001 '**' 0.01 '*' 0.05 '.' 0.1 ' ' 1
```

```r
# percent_coverage vs p++ B
mod20 =  aov(percent_coverage_p_plus_plus_given_plus_parameter_1 ~ genotype*age,
             data = all_params)
summary(mod20)
```

```
##                 Df Sum Sq Mean Sq F value   Pr(>F)
## genotype         1 0.0977 0.09770   8.916 0.003218 **
## age              2 0.2527 0.12637  11.533 1.93e-05 ***
## genotype:age     2 0.1645 0.08227   7.508 0.000737 ***
## Residuals      181 1.9833 0.01096
## ---
## Signif. codes:  0 '***' 0.001 '**' 0.01 '*' 0.05 '.' 0.1 ' ' 1
```

```r
# percent_coverage vs p++ A post hoc
ph37 = TukeyHSD(aov(percent_coverage_p_plus_plus_given_plus_parameter_0 ~ age+genotype,
                    data = all_params), which = "age")
ph37
```

```
##   Tukey multiple comparisons of means
##     95% family-wise confidence level
##
## Fit: aov(formula = percent_coverage_p_plus_plus_given_plus_parameter_0 ~ age + genotype, data = all_
##
## $age
##                           diff          lwr         upr     p adj
## Old-Middle Aged    -0.12905698 -0.187605529 -0.07050842 0.0000015
## Young-Middle Aged   0.05505998  0.002687646  0.10743232 0.0368025
## Young-Old           0.18411696  0.123941445  0.24429247 0.0000000
```

```r
ph38 = TukeyHSD(aov(percent_coverage_p_plus_plus_given_plus_parameter_0 ~ age+genotype,
                    data = all_params), which = "genotype")
ph38
```

```
##   Tukey multiple comparisons of means
##     95% family-wise confidence level
##
## Fit: aov(formula = percent_coverage_p_plus_plus_given_plus_parameter_0 ~ age + genotype, data = all_
##
## $genotype
##              diff        lwr        upr     p adj
## WT-KMO 0.05991316 0.02190046 0.09792587 0.0021722
```

```r
# percent_coverage vs p++ B post hoc
ph39 = TukeyHSD(aov(percent_coverage_p_plus_plus_given_plus_parameter_1 ~ age+genotype,
                    data = all_params), which = "age")
ph39
```

```
##   Tukey multiple comparisons of means
##     95% family-wise confidence level
##
## Fit: aov(formula = percent_coverage_p_plus_plus_given_plus_parameter_1 ~ age + genotype, data = all_p
##
## $age
##                            diff         lwr          upr     p adj
## Old-Middle Aged    0.06298365  0.01483197  0.111135329 0.0064995
## Young-Middle Aged -0.03564190 -0.07871412  0.007430314 0.1264003
## Young-Old         -0.09862555 -0.14811528 -0.049135824 0.0000146
```

```
ph40 = TukeyHSD(aov(percent_coverage_p_plus_plus_given_plus_parameter_1 ~ age+genotype,
                    data = all_params), which = "genotype")
ph40
```

```
##   Tukey multiple comparisons of means
##     95% family-wise confidence level
##
## Fit: aov(formula = percent_coverage_p_plus_plus_given_plus_parameter_1 ~ age + genotype, data = all_p
##
## $genotype
##              diff        lwr        upr     p adj
## WT-KMO 0.04366754 0.01240502 0.07493006 0.0064452
```

```
subtest10 =  manova(cbind(percent_coverage_p_plus_minus_given_plus_parameter_0,
                          percent_coverage_p_plus_minus_given_plus_parameter_1)
                    ~ genotype*age,
                    data = all_params)
summary(subtest10)
```

```
##                Df   Pillai approx F num Df den Df    Pr(>F)
## genotype        1 0.266729   32.738      2    180 7.478e-13 ***
## age             2 0.237716   12.208      4    362 2.545e-09 ***
## genotype:age    2 0.075372    3.544      4    362  0.007475 **
## Residuals     181
## ---
## Signif. codes:  0 '***' 0.001 '**' 0.01 '*' 0.05 '.' 0.1 ' ' 1
```

```
# percent_coverage vs p+- A
mod21 =  aov(percent_coverage_p_plus_minus_given_plus_parameter_0 ~ genotype*age,
             data = all_params)
summary(mod21)
```

```
##                Df Sum Sq Mean Sq F value  Pr(>F)
## genotype        1 0.0510 0.05103   4.959 0.02719 *
## age             2 0.5402 0.27008  26.246 9.8e-11 ***
## genotype:age    2 0.1343 0.06713   6.524 0.00184 **
## Residuals     181 1.8625 0.01029
## ---
## Signif. codes:  0 '***' 0.001 '**' 0.01 '*' 0.05 '.' 0.1 ' ' 1
```

```r
# percent_coverage vs p+- B
mod22 =  aov(percent_coverage_p_plus_minus_given_plus_parameter_1 ~ genotype*age,
             data = all_params)
summary(mod22)
```

```
##                Df Sum Sq Mean Sq F value   Pr(>F)
## genotype        1 0.0939 0.09392  12.742 0.000458 ***
## age             2 0.1481 0.07406  10.048 7.28e-05 ***
## genotype:age    2 0.0919 0.04595   6.234 0.002409 **
## Residuals     181 1.3341 0.00737
## ---
## Signif. codes:  0 '***' 0.001 '**' 0.01 '*' 0.05 '.' 0.1 ' ' 1
```

```r
# percent_coverage vs p+- A post hoc
ph41 = TukeyHSD(aov(percent_coverage_p_plus_minus_given_plus_parameter_0 ~ age+genotype,
                    data = all_params), which = "age")
ph41
```

```
##   Tukey multiple comparisons of means
##     95% family-wise confidence level
##
## Fit: aov(formula = percent_coverage_p_plus_minus_given_plus_parameter_0 ~ age + genotype, data = all_
##
## $age
##                           diff         lwr          upr     p adj
## Old-Middle Aged    0.09370933  0.04728215  0.140136517 0.0000112
## Young-Middle Aged -0.04639450 -0.08792414 -0.004864861 0.0243498
## Young-Old         -0.14010383 -0.18782114 -0.092386515 0.0000000
```

```r
ph42 = TukeyHSD(aov(percent_coverage_p_plus_minus_given_plus_parameter_0 ~ age+genotype,
                    data = all_params), which = "genotype")
ph42
```

```
##   Tukey multiple comparisons of means
##     95% family-wise confidence level
##
## Fit: aov(formula = percent_coverage_p_plus_minus_given_plus_parameter_0 ~ age + genotype, data = all_
##
## $genotype
##               diff         lwr          upr     p adj
## WT-KMO -0.03611208 -0.06625498 -0.005969191 0.0191405
```

```r
# percent_coverage vs p+- B post hoc
ph43 = TukeyHSD(aov(percent_coverage_p_plus_minus_given_plus_parameter_1 ~ age+genotype,
                    data = all_params), which = "age")
ph43
```

```
##   Tukey multiple comparisons of means
##     95% family-wise confidence level
##
## Fit: aov(formula = percent_coverage_p_plus_minus_given_plus_parameter_1 ~ age + genotype, data = all_
```

```
##
## $age
##                             diff          lwr          upr     p adj
## Old-Middle Aged   -0.04380650 -0.083040754 -0.004572249 0.0244448
## Young-Middle Aged  0.03196638 -0.003129101  0.067061855 0.0823837
## Young-Old          0.07577288  0.035448376  0.116097381 0.0000460
```

```
ph44 = TukeyHSD(aov(percent_coverage_p_plus_minus_given_plus_parameter_1 ~ age+genotype,
                    data = all_params), which = "genotype")
ph44
```

```
##   Tukey multiple comparisons of means
##     95% family-wise confidence level
##
## Fit: aov(formula = percent_coverage_p_plus_minus_given_plus_parameter_1 ~ age + genotype, data = all_
##
## $genotype
##               diff         lwr         upr     p adj
## WT-KMO -0.04342434 -0.06889721 -0.01795146 0.0009375
```

```
subtest11 =  manova(cbind(percent_coverage_p_plus_zero_given_plus_parameter_0,
                          percent_coverage_p_plus_zero_given_plus_parameter_1)
                    ~ genotype*age,
                    data = all_params)
summary(subtest11)
```

```
##               Df   Pillai approx F num Df den Df    Pr(>F)
## genotype       1 0.299410   38.463      2    180 1.235e-14 ***
## age            2 0.200394   10.078      4    362 9.605e-08 ***
## genotype:age   2 0.033664    1.549      4    362    0.1874
## Residuals    181
## ---
## Signif. codes:  0 '***' 0.001 '**' 0.01 '*' 0.05 '.' 0.1 ' ' 1
```

```
# percent_coverage vs p+0 A
mod23 =  aov(percent_coverage_p_plus_zero_given_plus_parameter_0 ~ genotype*age,
             data = all_params)
summary(mod23)
```

```
##               Df  Sum Sq  Mean Sq F value   Pr(>F)
## genotype       1 0.01708 0.017079  50.419 2.74e-11 ***
## age            2 0.01487 0.007436  21.951 2.91e-09 ***
## genotype:age   2 0.00122 0.000612   1.806    0.167
## Residuals    181 0.06131 0.000339
## ---
## Signif. codes:  0 '***' 0.001 '**' 0.01 '*' 0.05 '.' 0.1 ' ' 1
```

```
# percent_coverage vs p+0 B
mod24 =  aov(percent_coverage_p_plus_zero_given_plus_parameter_1 ~ genotype*age,
             data = all_params)
summary(mod24)
```

```
##                Df  Sum Sq   Mean Sq F value   Pr(>F)    
## genotype       1 0.00108 0.0010798  12.555 0.000503 ***
## age            2 0.00046 0.0002298   2.672 0.071817 .  
## genotype:age   2 0.00032 0.0001602   1.863 0.158184    
## Residuals    181 0.01557 0.0000860                     
## ---
## Signif. codes:  0 '***' 0.001 '**' 0.01 '*' 0.05 '.' 0.1 ' ' 1
```

```r
# percent_coverage vs p+0 A post hoc
ph45 = TukeyHSD(aov(percent_coverage_p_plus_zero_given_plus_parameter_0 ~ age+genotype,
                    data = all_params), which = "age")
ph45
```

```
##   Tukey multiple comparisons of means
##     95% family-wise confidence level
## 
## Fit: aov(formula = percent_coverage_p_plus_zero_given_plus_parameter_0 ~ age + genotype, data = all_
## 
## $age
##                            diff          lwr          upr     p adj
## Old-Middle Aged     0.018734374  0.010518088  0.026950660 0.0000007
## Young-Middle Aged -0.002401674 -0.009751234  0.004947886 0.7205682
## Young-Old         -0.021136048 -0.029580649 -0.012691446 0.0000000
```

```r
ph46 = TukeyHSD(aov(percent_coverage_p_plus_zero_given_plus_parameter_0 ~ age+genotype,
                    data = all_params), which = "genotype")
ph46
```

```
##   Tukey multiple comparisons of means
##     95% family-wise confidence level
## 
## Fit: aov(formula = percent_coverage_p_plus_zero_given_plus_parameter_0 ~ age + genotype, data = all_
## 
## $genotype
##               diff         lwr         upr p adj
## WT-KMO -0.01975412 -0.02508855 -0.01441969     0
```

```r
# percent_coverage vs p+0 B post hoc
ph47 = TukeyHSD(aov(percent_coverage_p_plus_zero_given_plus_parameter_1 ~ age+genotype,
                    data = all_params), which = "age")
ph47
```

```
##   Tukey multiple comparisons of means
##     95% family-wise confidence level
## 
## Fit: aov(formula = percent_coverage_p_plus_zero_given_plus_parameter_1 ~ age + genotype, data = all_
## 
## $age
##                             diff           lwr           upr     p adj
## Old-Middle Aged   -0.0042297361 -0.0083710429 -8.842925e-05 0.0440877
## Young-Middle Aged -0.0009203983 -0.0046248435  2.784047e-03 0.8272122
## Young-Old          0.0033093378 -0.0009470485  7.565724e-03 0.1604355
```

```r
ph48 = TukeyHSD(aov(percent_coverage_p_plus_zero_given_plus_parameter_1 ~ age+genotype,
                    data = all_params), which = "genotype")
ph48
```

```
##   Tukey multiple comparisons of means
##     95% family-wise confidence level
##
## Fit: aov(formula = percent_coverage_p_plus_zero_given_plus_parameter_1 ~ age + genotype, data = all_p
##
## $genotype
##                diff          lwr          upr     p adj
## WT-KMO -0.004655955 -0.007344703 -0.001967208 0.0007814
```

```r
subtest12 =  manova(cbind(percent_coverage_p_zero_plus_given_zero_parameter_0,
                          percent_coverage_p_zero_plus_given_zero_parameter_1)
                    ~ genotype*age,
                    data = all_params)
summary(subtest12)
```

```
##               Df   Pillai approx F num Df den Df   Pr(>F)
## genotype       1 0.031259   2.7750      2    172 0.065141 .
## age            2 0.049398   2.1906      4    346 0.069667 .
## genotype:age   2 0.094799   4.3041      4    346 0.002067 **
## Residuals    173
## ---
## Signif. codes:  0 '***' 0.001 '**' 0.01 '*' 0.05 '.' 0.1 ' ' 1
```

```r
# percent_coverage vs p0+ A
mod25 =  aov(percent_coverage_p_zero_plus_given_zero_parameter_0 ~ genotype*age,
             data = all_params)
summary(mod25)
```

```
##               Df Sum Sq Mean Sq F value   Pr(>F)
## genotype       1   2.12   2.116   2.524 0.113954
## age            2   5.08   2.538   3.027 0.051037 .
## genotype:age   2  13.50   6.750   8.050 0.000454 ***
## Residuals    173 145.05   0.838
## ---
## Signif. codes:  0 '***' 0.001 '**' 0.01 '*' 0.05 '.' 0.1 ' ' 1
## 8 observations deleted due to missingness
```

```r
# percent_coverage vs p0+ B
mod26 =  aov(percent_coverage_p_zero_plus_given_zero_parameter_1 ~ genotype*age,
             data = all_params)
summary(mod26)
```

```
##               Df Sum Sq Mean Sq F value   Pr(>F)
## genotype       1   2.63   2.628   4.614 0.033112 *
## age            2   3.05   1.526   2.679 0.071508 .
## genotype:age   2   8.94   4.470   7.848 0.000546 ***
## Residuals    173  98.54   0.570
```

```
## ---
## Signif. codes:  0 '***' 0.001 '**' 0.01 '*' 0.05 '.' 0.1 ' ' 1
## 8 observations deleted due to missingness
```

```r
# percent_coverage vs p0+ A post hoc
ph49 = TukeyHSD(aov(percent_coverage_p_zero_plus_given_zero_parameter_0 ~ age+genotype,
                    data = all_params), which = "age")
ph49
```

```
##   Tukey multiple comparisons of means
##     95% family-wise confidence level
##
## Fit: aov(formula = percent_coverage_p_zero_plus_given_zero_parameter_0 ~ age + genotype, data = all_
##
## $age
##                          diff          lwr       upr     p adj
## Old-Middle Aged   0.06326119 -0.365064742 0.4915871 0.9350488
## Young-Middle Aged 0.38050309 -0.008597305 0.7696035 0.0568050
## Young-Old         0.31724190 -0.127777514 0.7622613 0.2137684
```

```r
ph50 = TukeyHSD(aov(percent_coverage_p_zero_plus_given_zero_parameter_0 ~ age+genotype,
                    data = all_params), which = "genotype")
ph50
```

```
##   Tukey multiple comparisons of means
##     95% family-wise confidence level
##
## Fit: aov(formula = percent_coverage_p_zero_plus_given_zero_parameter_0 ~ age + genotype, data = all_
##
## $genotype
##              diff        lwr        upr     p adj
## WT-KMO -0.2091272 -0.4901603 0.07190596 0.1437262
```

```r
# percent_coverage vs p0+ B post hoc
ph51 = TukeyHSD(aov(percent_coverage_p_zero_plus_given_zero_parameter_1 ~ age+genotype,
                    data = all_params), which = "age")
ph51
```

```
##   Tukey multiple comparisons of means
##     95% family-wise confidence level
##
## Fit: aov(formula = percent_coverage_p_zero_plus_given_zero_parameter_1 ~ age + genotype, data = all_
##
## $age
##                           diff        lwr        upr     p adj
## Old-Middle Aged    0.04867761 -0.3039831 0.40133828 0.9430273
## Young-Middle Aged -0.26335427 -0.5837187 0.05701019 0.1297517
## Young-Old         -0.31203187 -0.6784371 0.05437332 0.1120660
```

```r
ph52 = TukeyHSD(aov(percent_coverage_p_zero_plus_given_zero_parameter_1 ~ age+genotype,
                    data = all_params), which = "genotype")
ph52
```

```
##   Tukey multiple comparisons of means
##     95% family-wise confidence level
##
## Fit: aov(formula = percent_coverage_p_zero_plus_given_zero_parameter_1 ~ age + genotype, data = all_p
##
## $genotype
##              diff         lwr       upr     p adj
## WT-KMO 0.2327797 0.001391984 0.4641673 0.0486525
```

```
subtest13 =  manova(cbind(percent_coverage_p_zero_zero_given_zero_parameter_0,
                          percent_coverage_p_zero_zero_given_zero_parameter_1)
                    ~ genotype*age,
                    data = all_params)
summary(subtest13)
```

```
##                 Df   Pillai approx F num Df den Df   Pr(>F)
## genotype         1 0.066927   6.1685      2    172 0.002587 **
## age              2 0.022379   0.9788      4    346 0.419080
## genotype:age     2 0.038059   1.6780      4    346 0.154577
## Residuals      173
## ---
## Signif. codes:  0 '***' 0.001 '**' 0.01 '*' 0.05 '.' 0.1 ' ' 1
```

```
# percent_coverage vs p00 A
mod27 =  aov(percent_coverage_p_zero_zero_given_zero_parameter_0 ~ genotype*age,
             data = all_params)
summary(mod27)
```

```
##                 Df Sum Sq Mean Sq F value Pr(>F)
## genotype         1  0.010 0.01032   0.127  0.722
## age              2  0.243 0.12135   1.495  0.227
## genotype:age     2  0.348 0.17415   2.146  0.120
## Residuals      173 14.040 0.08116
## 8 observations deleted due to missingness
```

```
# percent_coverage vs p00 B
mod28 =  aov(percent_coverage_p_zero_zero_given_zero_parameter_1 ~ genotype*age,
             data = all_params)
summary(mod28)
```

```
##                 Df Sum Sq Mean Sq F value Pr(>F)
## genotype         1  0.377  0.3771   6.730 0.0103 *
## age              2  0.143  0.0714   1.274 0.2823
## genotype:age     2  0.315  0.1574   2.809 0.0630 .
## Residuals      173  9.695  0.0560
## ---
## Signif. codes:  0 '***' 0.001 '**' 0.01 '*' 0.05 '.' 0.1 ' ' 1
## 8 observations deleted due to missingness
```

```
# percent_coverage vs p00 A post hoc
ph53 = TukeyHSD(aov(percent_coverage_p_zero_zero_given_zero_parameter_0 ~ age+genotype,
                    data = all_params), which = "age")
ph53
```

```
##   Tukey multiple comparisons of means
##     95% family-wise confidence level
##
## Fit: aov(formula = percent_coverage_p_zero_zero_given_zero_parameter_0 ~ age + genotype, data = all_p
##
## $age
##                            diff         lwr        upr     p adj
## Old-Middle Aged    0.03980876 -0.08922519 0.16884271 0.7464703
## Young-Middle Aged -0.05462131 -0.17183850 0.06259588 0.5144235
## Young-Old         -0.09443007 -0.22849296 0.03963282 0.2216197
```

```
ph54 = TukeyHSD(aov(percent_coverage_p_zero_zero_given_zero_parameter_0 ~ age+genotype,
                    data = all_params), which = "genotype")
ph54
```

```
##   Tukey multiple comparisons of means
##     95% family-wise confidence level
##
## Fit: aov(formula = percent_coverage_p_zero_zero_given_zero_parameter_0 ~ age + genotype, data = all_p
##
## $genotype
##               diff        lwr        upr     p adj
## WT-KMO -0.01845676 -0.1031185 0.06620498 0.6675365
```

```
# percent_coverage vs p00 B post hoc
ph55 = TukeyHSD(aov(percent_coverage_p_zero_zero_given_zero_parameter_1 ~ age+genotype,
                    data = all_params), which = "age")
ph55
```

```
##   Tukey multiple comparisons of means
##     95% family-wise confidence level
##
## Fit: aov(formula = percent_coverage_p_zero_zero_given_zero_parameter_1 ~ age + genotype, data = all_p
##
## $age
##                            diff         lwr        upr     p adj
## Old-Middle Aged   -0.05957449 -0.16719633 0.04804736 0.3923448
## Young-Middle Aged  0.01906356 -0.07870242 0.11682953 0.8895848
## Young-Old          0.07863804 -0.03317823 0.19045432 0.2226427
```

```
ph56 = TukeyHSD(aov(percent_coverage_p_zero_zero_given_zero_parameter_1 ~ age+genotype,
                    data = all_params), which = "genotype")
ph56
```

```
##   Tukey multiple comparisons of means
##     95% family-wise confidence level
##
## Fit: aov(formula = percent_coverage_p_zero_zero_given_zero_parameter_1 ~ age + genotype, data = all_p
##
## $genotype
##               diff        lwr         upr     p adj
## WT-KMO -0.08883348 -0.1594463 -0.01822064 0.0139747
```
